# The effects of 12-weeks resveratrol supplementation on cognition, gastrointestinal microbiota, and systemic inflammation, in an overweight and obese human population: a randomized, double-blind, placebo controlled, parallel groups trial

**DOI:** 10.3389/fnut.2026.1839709

**Published:** 2026-07-01

**Authors:** Emma Wightman, John Lodge, David Kennedy, Samantha Bowerbank, William Cheung, Lewis Cuthbertson, Andrew Nelson, Darren Smith

**Affiliations:** 1 NUTRAN at Northumbria University, Northumbria University, Newcastle upon Tyne, United Kingdom; 2 Centre for Health and Life Sciences Research, School of Human Sciences, London Metropolitan University, London, United Kingdom; 3 School of Geography and Natural Science, Northumbria University, Newcastle upon Tyne, United Kingdom; 4 NU-OMICS DNA Sequencing Research Facility, Department of Applied Sciences, Northumbria University, Newcastle upon Tyne, United Kingdom

**Keywords:** cognition, gastrointestinal microbiota, inflammation, obese, overweight, resveratrol

## Abstract

**Background:**

Resveratrol appears to offer greater cognitive benefit to compromised models, such as in type II diabetes mellitus, menopause, and high body mass index (BMI), relative to healthy cohorts. With regards high BMI, hypertension, insulin resistance, oxidative stress, and inflammation have been posited as mechanisms underpinning cognitive decrements, and recent advancements in gut-brain-axis research have linked high BMI with inflammation via gut dysbiosis. Polyphenols have been evidenced to act prebiotically in the gut, to mediate anti-inflammatory effects in animal models, and this presents a mechanism by which resveratrol could bolster cognition in high BMI individuals.

**Aims:**

The current study investigates whether resveratrol can confer cognitive benefit to individuals with a high BMI, and whether these effects coincide with changes in the gut microbiome, urinary metabolome and biological markers of adiposity (anthropomorphic and blood biomarkers) and inflammation/oxidation.

**Methods:**

*N* = 99 male and females (35–60 years, mean age 47.51 years), with a BMI between 25 and 42 kg/m^2^, received either 500 mg Veri-te™ resveratrol, or placebo, daily for 12 weeks. This supplementation period was bookended by visits to the laboratory for urine, blood, and stool sampling, and cognitive testing, which was assessed pre-and post-dose during both the acute and chronic testing visit.

**Results:**

Participants in the placebo control group presented with existing differences on cognitive outcomes at baseline, which makes interpretation of apparent improvements in this group relative to resveratrol, problematic. No significant differences were observed within or between groups on any microbiome, urinary metabolome, biological markers of adiposity or inflammation/oxidation markers.

**Conclusion:**

The absence of effects on the underlying biological mechanisms rationalized to underpin cognitive improvements in high BMI individuals likely explains the null results in the resveratrol intervention group. Effects attributed to the placebo control condition are explained as the persistence of pre-existing effects in this group of participants, and this may underlie the need to factor pre-enrolment aptitude into randomization in nutritional intervention trials. The lack of change in the gut microbiome of a healthy human cohort, following 12 weeks of resveratrol supplementation, is a positive indication, showing no deleterious disruption within this environment. Future studies may wish to investigate these effects in those with a disrupted gut microbiome.

**Clinical trial registration:**

The study was pre-registered on clinicaltrials.gov (identifier: NCT03448094).

## Introduction

Resveratrol (3,4′,5 trihydroxystilbene) is a phytoalexin polyphenol with over half a million research papers now dedicated to its investigation. It is unsurprising that resveratrol dominates the polyphenol literature, given the number of biological pathways that it can interact with and, of importance here, the proportion of these which are of relevance for brain function.

Arguably the most promising neural mechanism underpinning brain function, and cognitive function specifically, is the cerebral blood flow effects of resveratrol; effects attributed to the modulation of platelet aggregation (1), and enhancement of endothelium-dependent vaso-relaxation by promotion of nitric oxide synthase and/or nitric oxide synthesis (2). Our own lab has published multiple papers over the past 15 years evidencing the robust cerebral blood flow response to resveratrol supplementation (3–6). However, despite consistent increases in blood perfusion, observed in a range of models/methodologies, cognitive effects have been scarce and unconvincing, and this is in line with the appraisal of the wider resveratrol literature [e.g., (7)].

This lack of cognitive response may be due to the fact that the current literature is dominated by trials conducted in healthy participants, likely at the peak of their cognitive abilities; meaning the cerebral perfusion effects, and indeed other mechanisms, were simply not required. This is supported by research from other groups, where investigations into more compromised models do result in cognitive enhancement. Evans et al. (8), for example, report improved verbal memory in post-menopausal women [a life stage which is associated with disruption to many cognitive functions, and an often described general ‘brain fog’, due to oestrogen depletion (9)], following 14 weeks supplementation of a 75 mg daily dose. Wong et al. (10) observed a better cognitive performance index in adults with type II diabetes mellitus [where mild cognitive impairment is a greater risk factor due to chronic damage to, and dysfunction of, blood vessels, nerves, brain, and other tissues and organs (11)], and high body mass index (average 30.3 kg/m^2^) following 75- and 300 mg resveratrol, 75 min after consumption. And Witte et al. (12) found improved word retention following 26 weeks of 200 mg resveratrol/day in healthy overweight/obese adults (with a body mass index ranging 25–30 kg/m^2^).

Overweight and obese models provide a compromised model of cognition for several reasons. Low-grade inflammation is a characteristic of the overweight/obese body mass index, and adipose tissue releases many inflammatory mediators (13). This, alongside hypertension, insulin resistance, and oxidative stress, have been posited as likely mechanisms underpinning the cognitive disruption, particularly of executive function, which correlates positively with high body mass index (14). Recent advancements in gut-brain-axis research have linked high body mass index with inflammation via gut dysbiosis, hypothesizing that “inflammation is a matter of microbial dysbiosis resulting in obesity” (15). Further, these authors argue that prebiotics represent a viable restorative procedure for this gut dysbiosis, by promoting the growth and activity of beneficial microorganisms, as well as healthy dietary fibers that select for fermentative bacteria.

Polyphenols are proven to stimulate the growth of beneficial bacteria and, as such, are regarded as prebiotic compounds (16). As an example, supplementation with 494 mg daily cocoa flavanols, for 4 weeks, found significant increases in fermentative bacteria like bifidobacteria and lactobacilli, significantly decreased clostridia, and significant reductions in the plasma inflammatory markers triacylglycerol and c-reactive protein, in a group of N = 22 adults (17). Resveratrol specifically is also reported to beneficially modulate the composition and metabolite production of the human intestinal microbiota, as well as increasing the antioxidant capacity of the intestinal environment, as reported by Sampaio et al. (18) following a 48 h *in vitro* colonic fermentation with 150 mg resveratrol. And finally, in a mouse model of obesity-induced non-alcoholic fatty liver disease, 4 weeks supplementation with 100 mg/day resveratrol evinced prebiotic like effects against steatosis, inflammation and redox status, in line with those of the probiotic comparator *B. infantis*; efficacy which was enhanced when the two were combined (19).

Whilst there are currently no studies investigating the effects of resveratrol supplementation on the human microbiota, with regards any outcomes to the best of our knowledge, the above suggests that resveratrol could improve cognitive function by interacting with inflammatory pathways directly or indirectly resulting from being overweight/obese. Taken together, the current study aimed to investigate the effects of resveratrol on cognition in a group hypothesized to be compromised by systemic inflammation, caused by high body mass index. Given the above, this study additionally assesses the gut microbiome, urinary metabolome and biological markers of adiposity (anthropomorphic and blood biomarkers) and inflammation/oxidation.

## Methods

### Study design

A randomized, placebo-controlled, parallel groups design was utilized to compare the effects of 84 days of resveratrol supplementation, versus placebo, on cognition, mood, the gut microbiota, urinary metabolome, blood pressure, heart rate, body mass index and blood biomarkers [comprising markers of adiposity (cholesterol (total, low-, and high density lipoprotein), and triglycerides)], and oxidation/inflammation (C-reactive protein (CRP), Ferric Reducing Antioxidant Power (FRAP), and Interleukin-6 (IL-6)), as well as glucose, and circulating levels of resveratrol and its metabolites (resveratrol-3-O-D glucoside, resveratrol-3-0 sulfate, and resveratrol-4-O-D glucoronide). The study was performed in accordance with the ethical principles that have their origin in the Declaration of Helsinki (1996), and with ethical approval granted by the University of Northumbria Department of Psychology (now known as School of Psychology) Ethics Committee, UK (reference number 1147). The trial was conducted in compliance with Good Clinical Practice (GCP) regulatory requirements.

### Determination of sample size

Cognition represented the primary outcome measure for this trial, with six measurements of this across the course of the study (one pre-dose, and two post-dose assessments on day 1, and on day 84). Assuming a conservatively small effect size (Cohen’s *f* = 0.25), requiring a minimum power of 0.9, and applying a statistical threshold of 0.05 to a between-factors ANOVA analysis, G*Power (20) estimated the sample size to be *N* = 102 for a 2-group design.

### Study population

**Figure 1:**
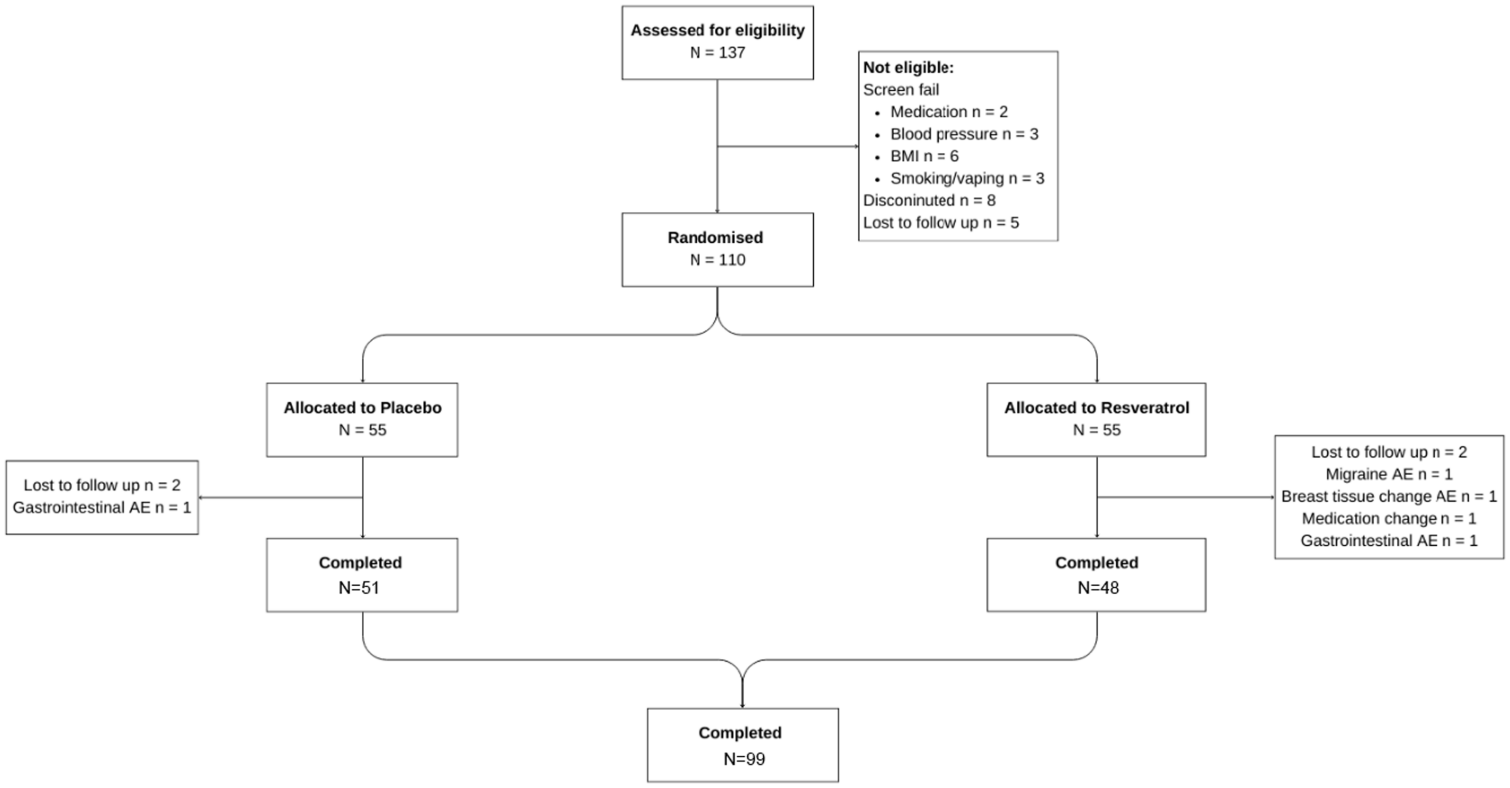
CONSORT diagram. Depicting participant disposition through the trial.

A sample of 137 male and female volunteers, aged 35–60 years (with a mean age 47.44 years), were initially recruited, and *N* = 110 were randomized into the trial. Eleven participants discontinued their participation following the initial testing visit, resulting in 99 participants completing the intervention (*N* = 76 female, *N* = 23 male). The full participant disposition through the trial, including reasons for the abovementioned discontinuations, are included in Figure 1. The participant characteristics for all *N* = 99 participants are found in Table 1.

**Table 1:** Participant mean (and Standard Deviations (SD), in italics) characteristics for *N* = 99 participants.

Measure	Mean (and *Standard deviation*)		
	Overall (*N* = 99)	Resveratrol (*N* = 51)	Placebo (*N* = 48)
Age (years)	47.51 (range 35–60) (*7.38*)	47.63 (range 36–60) (*7.21*)	47.38 (range 35–59) (*7.64*)
Education (years)	16.68 (*3.50*)	16.29 (*3.31*)	17.08 (*3.68*)
Sex	76F, 23 M	39F, 12 M	37F, 11 M
Race	97 W, 2A	50 W, 1A	47 W, 1A
Fruit and Veg consumption (portions/day)	3.30 (*1.19*)	2.96 (*1.19*)	3.67 (*1.09*)
Vegetarian/Pescetarian	3/1	2/1	0/1
Caffeine consumption (mg/day)	229.06 (*124.07*)	238.96 (*110.76*)	218.54 (*137.21*)
Systolic BP (mm/Hg)	130.12 (*13.18*)	130.81 (*14.23*)	129.38 (*12.07*)
Diastolic BP (mm/Hg)	85.18 (*9.74*)	86.01 (*9.12*)	84.29 (*10.39*)
Heart rate (beats per minute)	73.03 (*11.98*)	73.53 (*10.56*)	72.50 (*13.42*)
BMI (kg/m2)	30.29 (*4.29*)	30.22 (*4.16*)	30.36 (*4.46*)
Waist to hip ratio	0.90 (0*.07*)	0.91 (0*.07*)	0.89 (0*.06*)

With regards sex, F=Female, and M = male. With regards race, W = white, and A = Asian. N/A = not applicable. Table reports vegetarian and pescatarian only. All remaining participants selected ‘omnivore’. Analyses of the baseline characteristics between groups produced no significant differences.

The following inclusion criteria were applied to participants:

#### Inclusion criteria

- Participants self-report that they are in good health
- Participants are aged 35–60 years (inclusive)
- Have a Body Mass Index (BMI) of between 25–42 kg/m^2^
- Are willing to consume their normal diet during the 12-week supplementation period

A full list of the exclusion criteria can be found in the Supplementary materials.

### Treatment

Participants were randomly assigned via Latin square into one of two treatment conditions, and consumed two capsules daily:

1. 500 mg Veri-te™ resveratrol
2. Placebo (cellulose microcrystalline)

Each resveratrol (Veri-te™) capsule contained 250 mg of >98% pure synthetic trans-resveratrol. Placebo capsules comprised cellulose microcrystalline. The Veri-te™ resveratrol capsules were manufactured under current Good Manufacturing Practise (cGMP) and Hazard Analysis and Critical Control Points (HACCP) based food safety conditions. The manufacturer (Evolva SA – Basel, Switzerland) provided the treatments, which were identical in appearance (white vegetarian capsules); ensuring that both the research team and participants remained blind to the treatment condition. The lead researcher prepared both treatments into identical white bottles, containing 90 capsules in each (with the first bottle dispensed on Day 1, and the second at a 6-week check-up appointment) and, to ensure that blinding was maintained throughout the trial, a third-party researcher coded the treatments as A and B, and created a stratified randomization schedule.

Participants consumed their first (acute: Day 1) and final (chronic: Day 84 +/− 5 days) treatments in the lab, consuming the full dose (i.e., 2 capsules, providing the daily 500 mg dose). During the interim supplementation period, participants consumed one capsule in the morning and one in the evening. These were advised to be consumed 30 min after their breakfast and evening meals, respectively.

Compliance was primarily measured by a count of the returned capsules, and a treatment diary was used as a secondary compliance measure.

### Study outcome measures

#### Physiological measures

- Blood Pressure and Heart Rate (BP/HR): Measured using a fully automatic oscillometric device: Boso Medicus Blood Pressure monitor (Bosch + Sohn GmbH u. Co. KG) following standardized procedures
- Body Mass Index (BMI): Calculated using standard formula: weight (kg) / [height (m)]^2^

#### Mood

- Bond-Lader Visual Analogue Scales (21): Participants were required to respond to sixteen 100 mm line visual analogue scales, that are anchored with antonyms at either end (e.g., “alert-drowsy”). Outcomes comprise three factor analysis derived scores: “Alertness,” “Calmness” and “Contentment.”
- Profile Of Mood States (POMS) (22): The POMS required participants to respond based on how they “are feeling right now” to a list of 65 words and statements describing their feelings. Participants respond on a 5-point Likert scale, which provides six scale scores: Anger-Hostility, Confusion-Bewilderment, Depression-Dejection, Fatigue-Inertia, Tension-Anxiety and Vigor-Activity. In addition, a total mood disturbance score was calculated.

#### Cognitive task battery

Cognitive assessments were delivered using the Computerized Mental Performance Assessment System (COMPASS). This proprietorial battery of cognitive assessments has been in use within Northumbria University for almost 20 years, and is currently also used within a number of UK, US, New Zealand, and Australian Universities, companies and research organizations.

**Figure 2:**
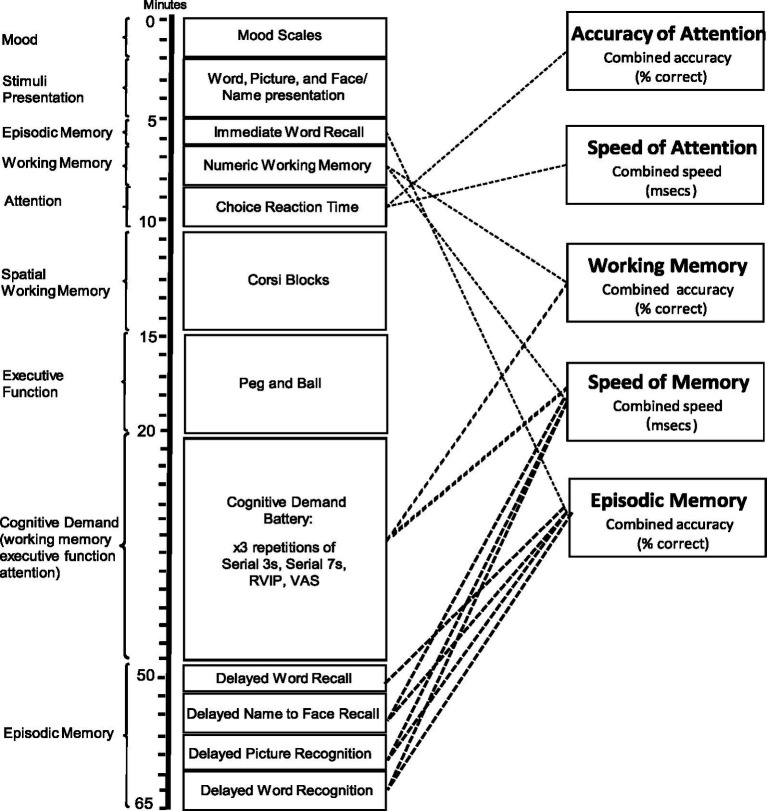
COMPASS cognitive task battery. Cognitive task order is listed centrally. The broad cognitive domain of each task is listed to the left. Global cognitive measures, which individual task outcome measures can be collapsed into, are listed on the right.

Figure 2 outlines the individual tasks used, the order in which they were presented, the approximate timings, the primary cognitive domain of each task (left-hand-side of the diagram), and the collapsible global outcome measures (right-hand-side). The cognitive tasks assessed visual/associative memory, verbal memory, spatial and working memory, executive function, attention, and cognitive demand. A full description of each task, and information detailing how the global outcome measures were calculated, can be found in (23).

#### Gut microbiota

Stool samples were collected at home by participants within 18 h of attending the research center, using Fe-Col® Faecal Collection Kits (Alpha Laboratories). Upon arrival at the laboratory, samples were immediately frozen at −80 °C, until sample preparation and analysis was conducted upon completion of the study. Here, each stool sample was partially defrosted, 100 mg weighed out, and DNA extracted using Qiagen HTP Power Soil DNA extraction kit, as per manufacturer’s instructions. All DNA was quantified using a Qubit, fluorimeter with DNA purity > 1.8 A260/A280. Amplification of the V4 region of the 16S rRNA gene using the method set out by (24).

The QIIME2 bioinformatics pipeline (25), was utilized to transform raw data files to amplicon sequence variants (ASVs) as described by (26). Data decontamination, analysis and visualizations were carried out in R studio, using decontam, phyloseq, vegan, ggplot2, gridextra, and scales packages.

A total of 8,530,597 reads, made up of 7,375 taxa, were screened for non-bacterial sequences and reduced to a total of 8,281,908 reads, containing 7,029 bacterial taxa. Samples were then screened for contaminants based on the prevalence of bacteria in negative control samples, further reducing the total library size to 5,035,953 and total number of bacterial taxa to 6,272 across 162 samples. Negative controls were then parsed from the dataset. Counts of bacterial taxa within samples were then normalized by conversion to relative abundance to account for variation in sequencing depth. The average read count for decontaminated samples was 31086.13. The SD was 17673.37 reads. Rarefaction curves were plotted to show sufficient sampling depth had been achieved.

Alpha diversity metrics were calculated and significance of multiple continuous variables determined using the Pairwise Wilcoxon test with Bonferroni adjustment. Beta diversity was assessed using weighted Bray-Curtis distance and displayed using PCoA analysis. PERMANOVA was used to determine significance of dissimilarity between groups. Differential abundance analyses were carried out in DeSeq2, which fits negative binomial generalized linear models between groups, and tests for significant difference using the Wald test, whilst controlling FDR using the Benjamini-Hochberg method.

#### Urinary metabolome

Spot urine samples, avoiding the first morning void, were provided pre-treatment consumption on each testing visit. Samples were collected in sterile 30 mL tubes, refrigerated, and 1 mL aliquots pipetted into sterilized microtubes and stored at −20 °C until analysis.

Samples were defrosted on ice, vortexed, and then an equal volume (100 μL) of urine was mixed with chilled (−20 °C) LC–MS-grade methanol. Samples were vortex mixed and chilled on ice for 30 min. Samples were then centrifuged at 13,000 rpm for 2 min. The top 150 μL was aliquoted, filtered and transferred to an LCMS vial for analysis. Quality control samples were prepared by aliquoting 5 μL of each sample together, vortexing and collecting 100 μL.

Hydrophilic interaction liquid chromatography (HILIC) based analysis was conducted using a Dionex 300 Ultra High-Pressure Liquid chromatography (UHPLC) and Q-Extractive high resolution mass spectrometer system. Samples were analyzed in a random order, with pooled quality control samples and blank injections. The data was acquired on both Positive and Negative mode polarity (independently). Thorough analysis methodology is detailed within (27).

#### Blood biomarkers

Intravenous blood samples were collected using 10 mL serum vacutainers to assess the following biomarker outcomes: total cholesterol, low-density lipoprotein (LDL), high-density lipoprotein (HDL), triglycerides, C-reactive protein (CRP), interleukin-6 (IL-6), ferric reducing antioxidant power (FRAP), glucose, resveratrol, resveratrol-3-O-D-glucoside, resveratrol-3-0-sulfate, and resveratrol-4-O-D-glucoronide. On both testing visits, samples were collected before the administration of the day’s treatment (fasted, pre-dose sample) and then at ~ 305 min post dose. Samples were inverted six times, and allowed to coagulate at room temperature for 1–2 h. Samples were processed within 2 h of collection. The samples were centrifuged at 600 RCF for 20 min at 4 °C to obtain serum, which was then pipetted into eppendorfs and stored at −80 °C until analysis.

Samples were thawed and then vortexed and sonicated for 5 min. In a microcentrifuge tube, 200 μL of sample was mixed with 900 μL of 0.1% formic acid in ethanol and 100 μL of naringenin. Samples were vortexed and sonicated prior to being centrifuged for 10 min at 17,000 g. The supernatant was removed and transferred into a fresh microcentrifuge tube. The remaining pellet was extracted with 1.2 mL of 83% aqueous ethanol using the procedure above, then both extracts were evaporated to dryness using a sample concentrator. The second extract was reconstituted in 70 μL of ethanol, of which 50 μL was transferred into the first extract, and 20 μL of taxifolin was added. The solution was then centrifuged and the supernatant transferred to an autosampler vial and 10 μL was analyzed via LC–MS/MS.

LC–MS analysis was performed using a Thermo Scientific® surveyor HPLC, consisting of an MS pump, autosampler and column oven, coupled to a Thermo Scientific® LTQ XL linear ion trap mass spectrometer (Thermo Scientific, Hemel Hempstead). Chromatographic separation was achieved using an Eclipse Plus™ C18 (100 × 4.6 mm, 3.5 μm) column (Agilent, Cheadle), using a gradient mobile phase consisting of A: Water + 0.1% formic acid and B: Methanol + 0.1% formic acid.

The mass spectrometer was optimized by auto tuning the MS parameters for resveratrol and was operated in negative selected reaction monitoring mode utilizing scan event.

### Procedure

Participants attended Northumbria University, UK, on four occasions. Firstly, during an initial screening/training visit, participants were informed of the study requirements, provided written informed consent and demographic information, were screened against the inclusion and exclusion criteria, and completed training on the cognitive and mood assessments.

Participants then attended the laboratory at 8.00 a.m on two separate occasions [Day 1 and Day 84 (+/− 5 days)]. At these visits, participants arrived after an overnight fast, and having refrained from alcohol for 24 h and caffeine for 18 h. Participants were required to complete a 4-day food diary prior to each visit and were instructed to consume the same food in these timeframes, prior to both visits. The food diaries were used simply to aid participants in maintaining this similar diet prior to testing sessions, and was not interpreted in any way by the research team.

**Figure 3:**
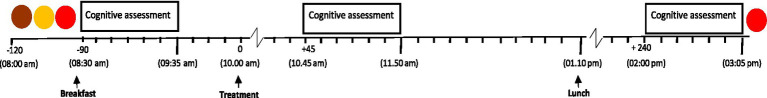
Day 1 and day 84 testing session timeline. Brown, yellow, and red markers represent stool, urine, and blood samples, respectively.

**Figure 4:**
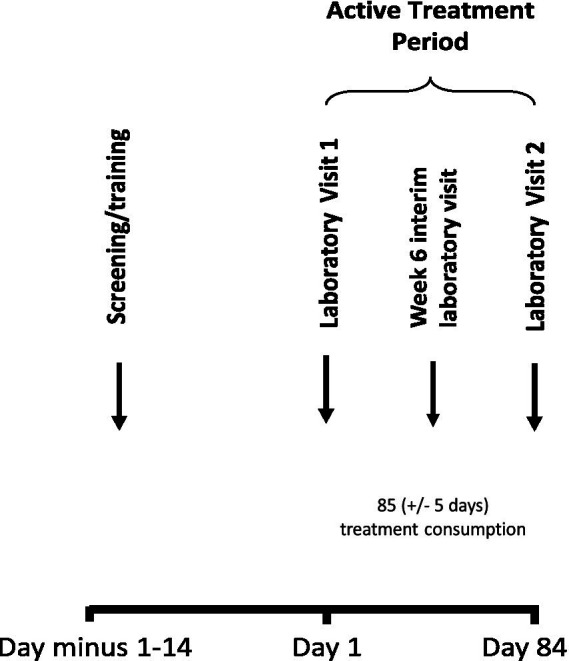
Overall study timeline.

The assessment procedure of each testing day was identical. Participants provided a stool sample that was collected either the previous day (no more than 18 h prior), or that morning, along with a urine sample (avoiding the first urination of the day). An intravenous blood sample was taken by a trained phlebotomist, and participants then consumed a standardised breakfast of toast and a decaffeinated tea or coffee (details of participants meals is included within Supplementary materials). At 8.30 a.m. participants began their baseline cognitive assessment; firstly completing the POMS, followed by the COMPASS battery, and a blood pressure reading was subsequently taken. Participants were visually isolated from each other for each of the cognitive assessments. All participants consumed their treatment at 10:00 a.m. Two further cognitive assessments and blood pressure readings commenced at 45- (10:45 a.m.) and 240- (2:00 p.m.) minutes post dose. Participants received a standardized lunch (see Supplementary materials for description) at 1:10 p.m. (190 min post dose). A final blood sample was collected at the end of each testing session at 3:05 p.m. (305 min post dose). At the end of Day 84, treatment diaries and returned capsules were assessed to confirm compliance. The timeline and assessments during Day 1 and Day 84 are shown in Figure 3, and an overall trial diagram can be found in Figure 4.

At the end of Day 1 participants took away a 6-week supply of their daily intervention and a diary to record their consumption of the treatment. After 6 weeks, participants returned to collect a further 6 weeks supply of treatment and confirm continued compliance with the study procedures and inclusion/exclusion criteria with the researcher.

### Statistical analysis

The statistical analyses for all outcome measures (aside from the stool and urine analyses, which are outlined in the method section above) were approached in the same way. The first analysis was to determine whether any true baseline differences existed between participants in the placebo group and the resveratrol group. This was first achieved by creating box and whisker plots on visit 1, pre-dose baseline data, for all individual outcome measures, to provide a visual observation. Secondly, independent samples t-tests compared the treatment groups at this pre-dose baseline time-point for all outcome measures. Unexpectedly, this approach did result in the observation of significant baseline differences between the treatment groups on many of the outcome measures, and this resulted in adapting the pre-registered statistical analysis plan for this trial (see below for description).

The next step in the pre-registered statistical analysis plan was to determine whether any pre-dose differences existed between treatment groups on visit 2. Again, independent samples *t*-tests compared the treatment groups at the pre-dose baseline time-point on visit 2. Effects here, following 12-weeks of supplementation, but before the final dose was consumed that day, would indicate pure chronic effects of the investigational product; i.e. the cumulative effects of 12-weeks supplementation.

The third stage of the analysis plan was to convert all post-dose data (data from the post-dose time-points during visit 1, pre-dose on visit 2, and post-dose on visit 2) to change-from-baseline, which was achieved by subtracting the visit 1 baseline scores from these post-dose time-points. This change-from-baseline data then compared the two treatment groups via repeated measures ANOVA, applying a within subjects factor of ‘time’ (which differed between the outcome measures, depending on how many times they were repeated within the study), and a between subjects factor of ‘treatment’ (which had two levels; resveratrol versus placebo). Effects here would indicate acute effects of treatment within visit 1 and/or within visit 2.

As mentioned above, given the widespread significant baseline differences between the treatment groups, this pre-registered approach was adapted to better accommodate this dataset. Specifically, on the Cholesterol, Serial 3 Subtraction ‘Total’, and ‘Correct’, and Rapid Visual Information Processing ‘Percentage Correct’, and ‘False Alarms’ outcome measures, where significant visit 1, pre-dose baseline differences were observed, we utilized the box and whisker plot-derived quartile ranges to create 3 ‘quartile groups’ within each treatment group. This created ‘high’ performers (i.e., those performing at or above their treatment groups upper quartile range), ‘low’ performers (i.e., those performing at or below their treatment groups lower quartile range), and ‘mid’ performers (those remaining participants). ‘Quartile group’ was then included as an additional between-subjects factor (alongside ‘treatment’) for these specific outcome measures.

All comparisons were Bonferroni corrected, and the results report means and standard error (SE) as the measure of variance. Observed power, in the form of Cohens *D*, is reported in brackets following each statistical reporting.

## Results

Results with significant treatment-related effects only are reported here. For completeness, the Supplementary materials reports all outcome results.

### Physiological measures

**Figure 5:**
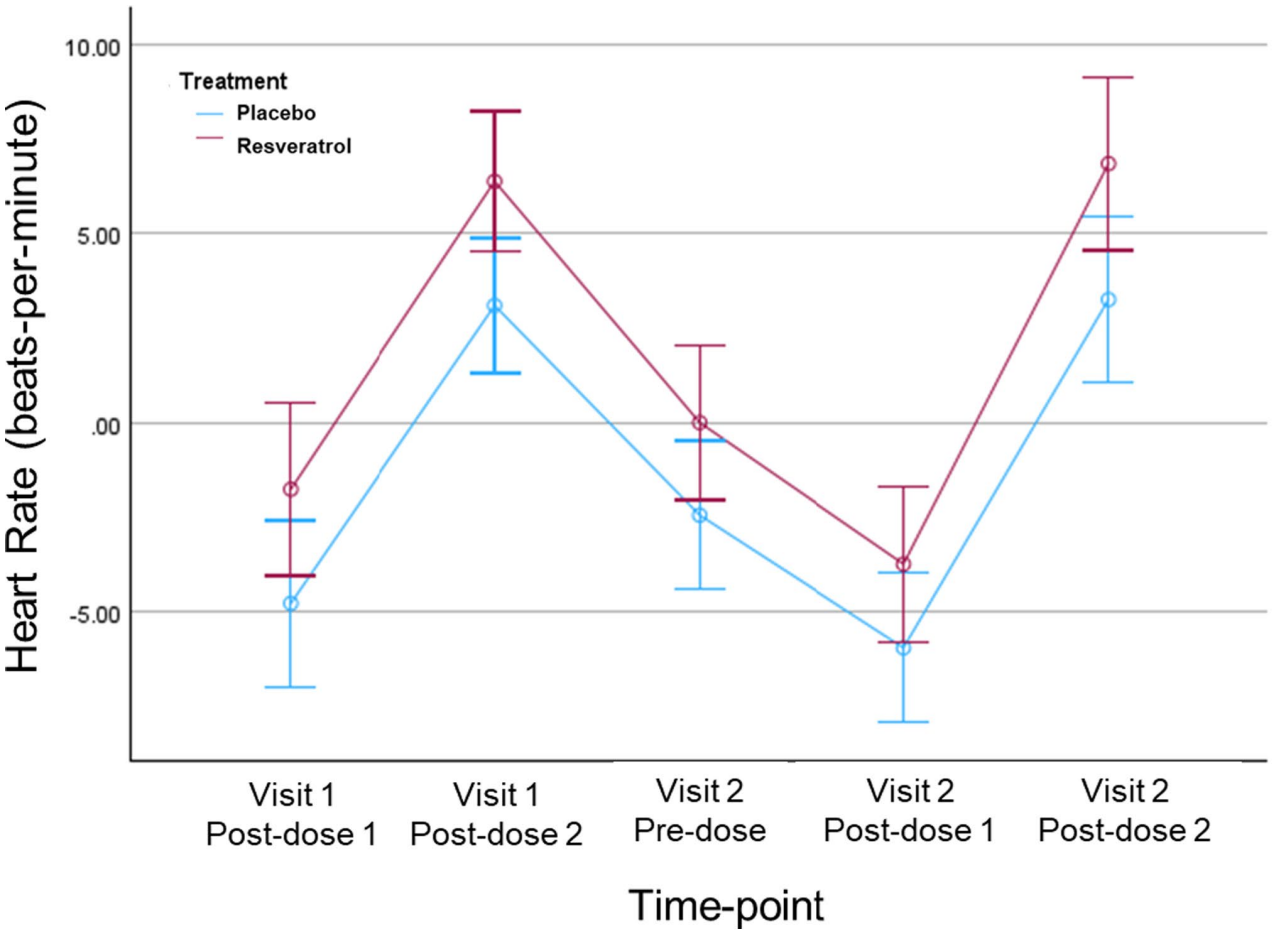
Heart rate change-from-baseline results.

- Blood pressure and heart rate (BP/HR):

Box and whisker plots (see Supplementary materials) identified 2 extreme outliers for visit 1, pre-dose baseline heart rate levels, both in the placebo condition. Following removal of these datasets, a significant main effect of treatment was observed; *F*(1,94) = 6.49, *p* = 0.01 (Cohens *D*; 0.71). Here, heart rate was significantly lower overall, relative to visit 1 baseline, in the placebo condition [mean change; −0.96 beats-per-minute (SE; 0.69)], compared to resveratrol [mean change; +1.55 beats-per-minute (SE; 0.75)]. See Figure 5 for graph depicting heart rate.

### Mood

**Figure 6:**
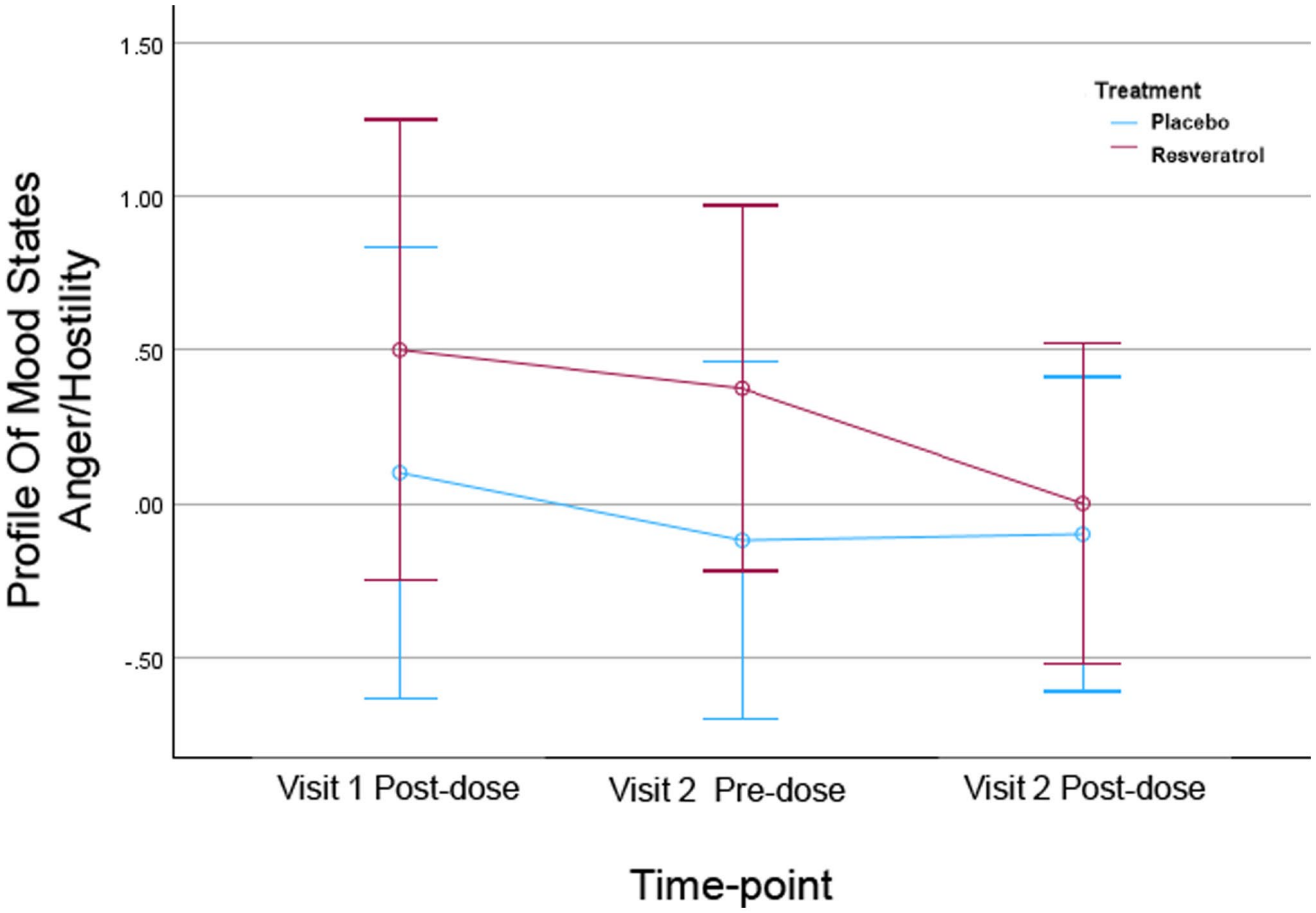
Profile of mood states ‘anger/hostility’ change-from-baseline results.

- Profile of mood states (POMS):

A single significant mood effect was observed on the POMS, and this revealed that those in the placebo condition reported a significantly smaller change in ‘anger/hostility’ pre-dose at visit 2 [0.60 (SE; 0.16)], as compared to those in the resveratrol condition [1.48 (SE; 0.35)]; t(96) = −2.35, *p* = 0.02 (Cohens *D*; −0.47). See Figure 6 for POMS ‘Anger/Hostility’ graph.

#### Cognitive task battery

- Serial 3 subtractions ‘errors’:

A single significant baseline difference on Serial 3 subtraction ‘errors’ was observed at repetition 1 during visit 2. Here, participants consuming placebo made approximately 1 fewer errors [1.30 (SE; 0.25)], compared to those consuming resveratrol [2.21 (SE; 0.34)]; t(92) = −2.17, *p* = 0.03 (Cohens *D*; −0.45).

**Figure 7:**
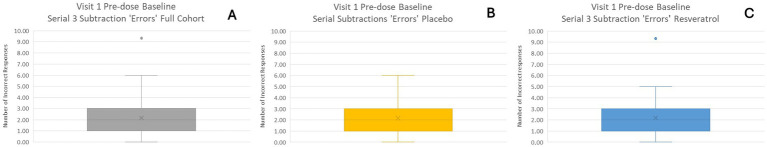
Box and whisker plot observations on visit 1, pre-dose baseline data for serial 3 subtraction ‘errors’ for the full cohort **(A)**, placebo condition **(B)**, and resveratrol condition **(C)**.

**Figure 8:**
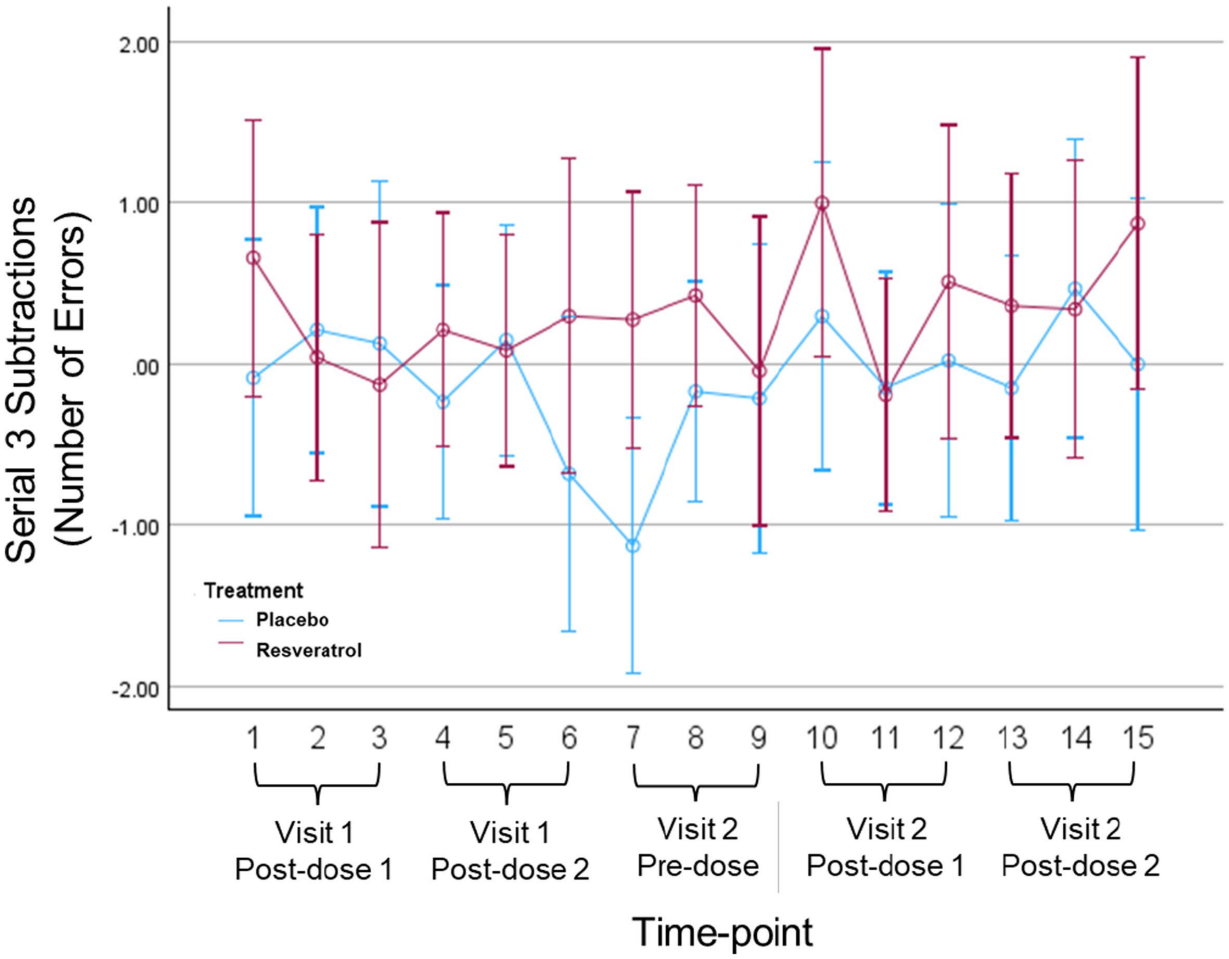
Serial 3 subtractions ‘errors’ change-from-baseline results.

However, whilst there was no statistically significant difference between treatment groups at the visit 1, pre-dose baseline time-point, reference to the box and whisker plot information from this time-point does reveal that a salient outlier in the resveratrol condition could be skewing interpretation of the overall effect here (See Figure 7C for resveratrol condition outlier, and Figures 7A,B for the full cohort, and the placebo condition, respectively). It is worth noting that this error response (approximately 9 errors) is still within the normal range of performance for this task, and so there was no justification to remove this datapoint from analysis. See Figure 8 for Serial 3 Subtractions ‘errors’ graph.

- Serial 7 subtractions ‘total’:

A significant baseline difference was observed on the 3rd, and final, pre-dose repetition during visit 2. Here, participants in the placebo condition completed significantly more subtractions [26.84 (SE; 1.56)] than those consuming resveratrol [22.31 (SE; 1.53)]; *t*(89) = 2.06, *p* = 0.04 (Cohens *D*; 0.43).

**Figure 9:**
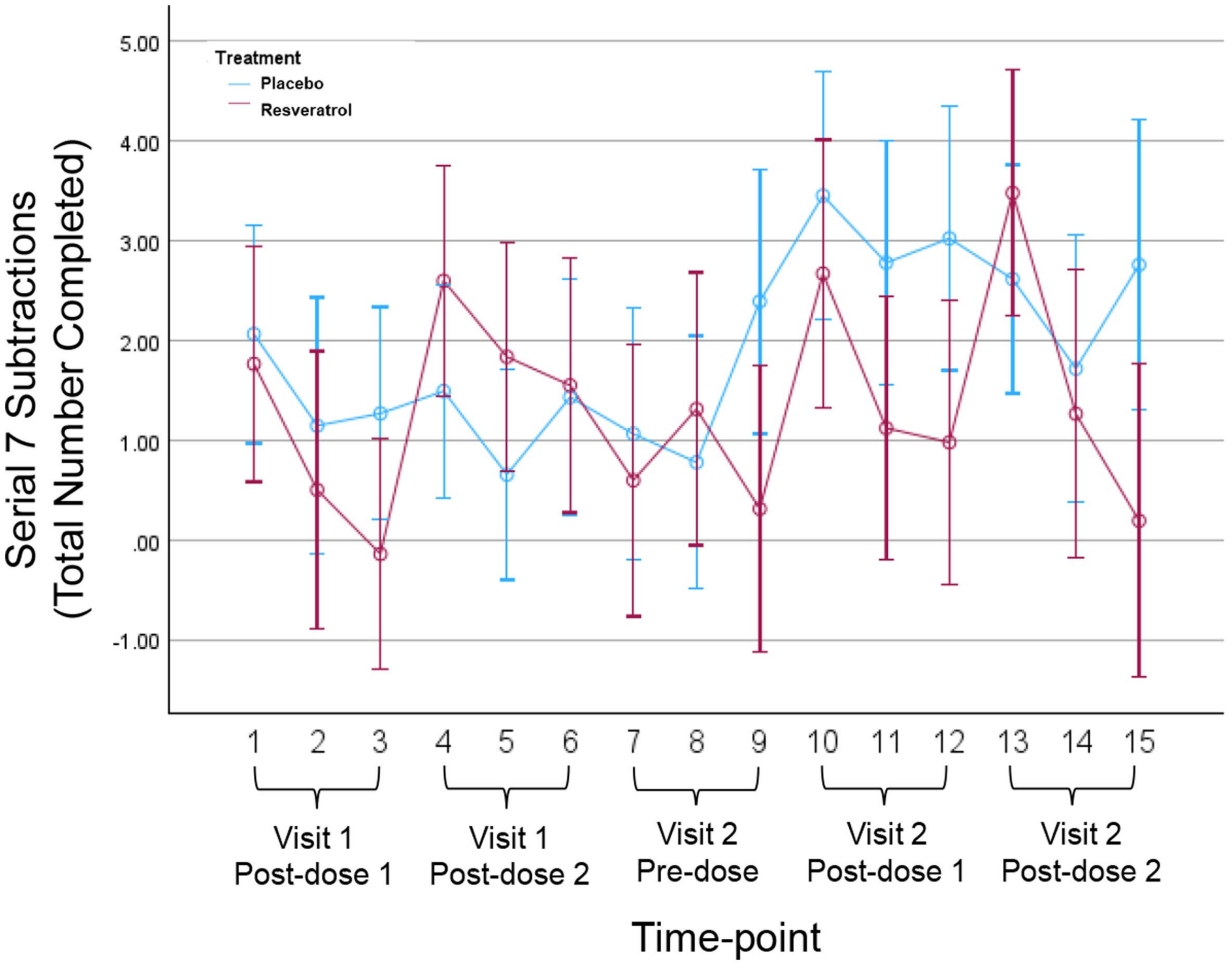
Serial 7 subtractions ‘total’ change-from-baseline results.

However, whilst there was no statistically significant difference between treatment groups at the visit 1, pre-dose baseline time-point, reference to the box and whisker plot information from this time-point does reveal stark differences between performance (see Table 2), which should be considered when interpreting this effect. See Figure 9 for Serial 7 Subtractions ‘Total’ graph.

- Rapid visual information processing (RVIP) ‘false alarms’:

**Table 2:** Box and whisker plot observations on visit 1, pre-dose baseline data for serial 7 subtraction ‘total’.

Condition	Median	Upper quartile	Lower quartile
Full cohort	22.93	28.67	16.00
Resveratrol	21.17	26.75	14.00
Placebo	24.43	32.00	16.67

This task outcome measure revealed significant differences at the visit 1, pre-dose baseline time-point; *t*(83) = −2.17, *p* = 0.03 (Cohens *D*; *−*0.47), with participants in the resveratrol condition making significantly more errors on this task [4.25 (SE; 0.81)] than those in the placebo group [2.42 (SE; 0.34)]. Reference to the box and whisker plots further evidences these preexisting differences between participants (see Table 3).

**Table 3:** Box and whisker plot observations on visit 1, pre-dose baseline data for rapid visual information processing ‘false alarms’.

Condition	**Median**	**Upper Quartile**	**Lower Quartile**
Full cohort	3.70	4.33	0.67
Resveratrol	4.69	6.17	0.75
Placebo	2.82	3.83	0.67

**Figure 10:**
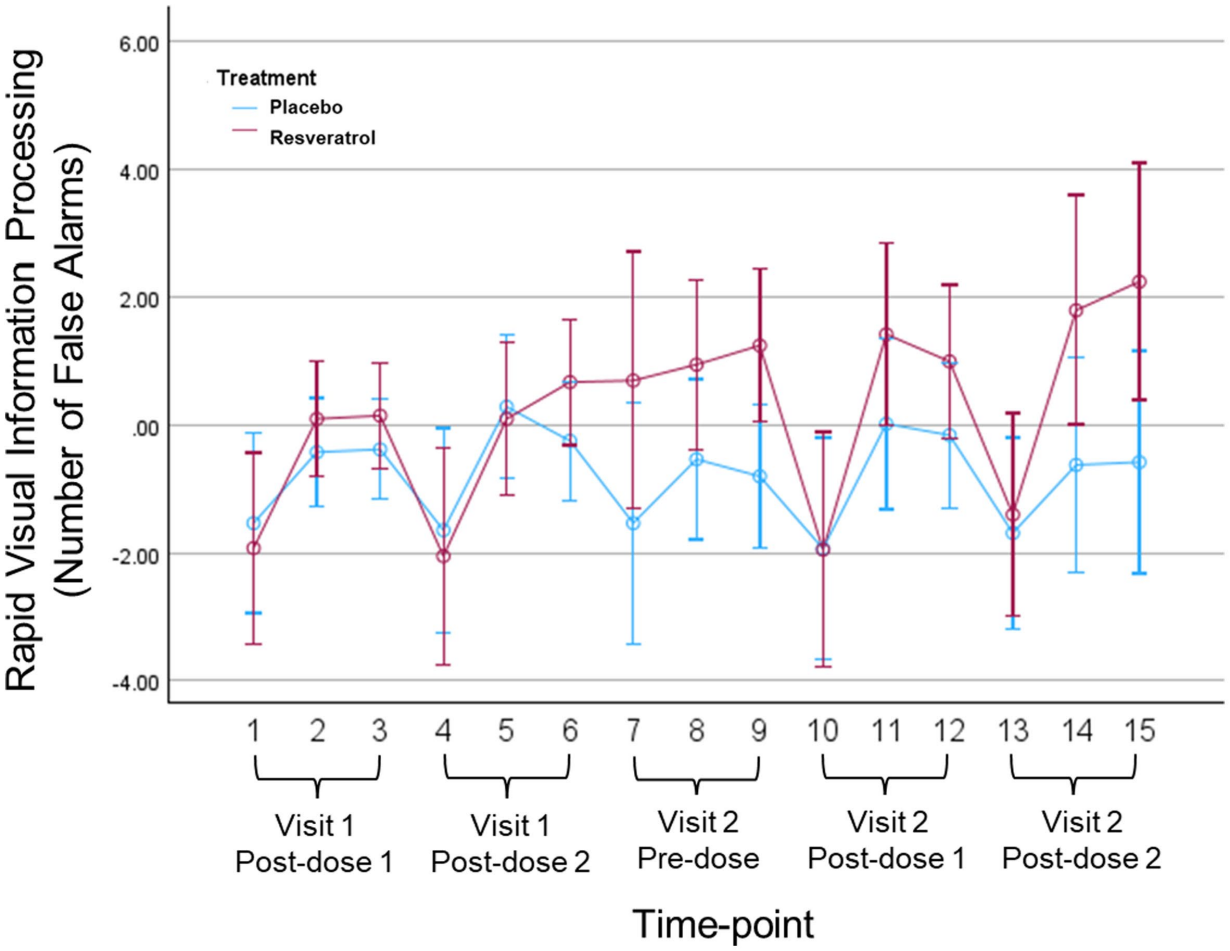
Rapid visual information processing ‘false alarm’ change-from-baseline results.

Given this preexisting difference between the treatment groups, the analysis plan for this task outcome measure incorporated ‘quartile group’ as an additional between-subjects factor in the repeated measure ANOVA analysis of change-from-baseline data. Here, only a significant interaction between treatment*time was observed: *F*(14,1,106) = 1.83, p = 0.03 (Cohens’ *D*; 0.93). However, when explored further, it was clear that this was caused by the quartile group to which participants belonged. A multivariate analysis, assessing treatment (the fixed factor) effects at all 15 timepoints, incorporating quartile group as a covariate, now observed no significant effect of treatment; *F*(15,68) = 1.02, *p* = 0.44; Wilk’s *Λ* = 0.82, partial n2 = 0.18 (Cohens *D*; 0.59). This was replaced by a significant effect of quartile group; *F*(15,68) = 2.94, *p* = <0.01; Wilk’s Λ = 0.61, partial n2 = 0.39 (Cohens *D*; 0.99). See Figure 10 for Rapid Visual Information Processing ‘False Alarm’ graph.

**Figure 11:**
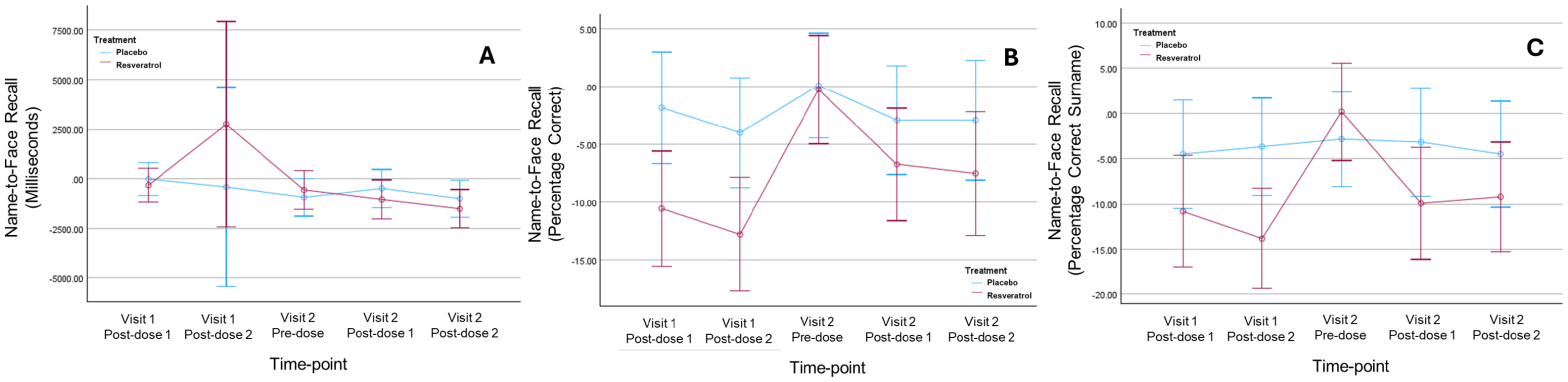
Name-to-face recall ‘reaction time’ **(A)**, ‘percentage correct’ **(B)**, and ‘Percentage Correct Surname’ **(C)** change-from-baseline results.

- Name-to-face recall ‘reaction time’:

A single significant pre-dose difference was observed at visit 2 for ‘reaction time’ on the name-to-face recall task. Here, those consuming placebo were significantly faster [11243.56 msec (SE; 483.69)] than those consuming resveratrol [12683.86 msec (SE; 434.18)]; t(95) = −2.21, *p* = 0.03 (Cohens *D*; −0.45). However, whilst there was no statistically significant difference between treatment groups at the visit 1, pre-dose baseline time-point, reference to the box and whisker plot information from this time-point does reveal stark differences between performance (see Table 4), which should be taken into account when interpreting this effect. See Figure 11A, for Name-to-Face Recall ‘Reaction Time’ graph.

- Name-to-face recall ‘percentage correct’:

**Table 4:** Box and Whisker Plot Observations on Visit 1, Pre-dose Baseline Data for Name-to-Face Recall ‘Reaction Time’ (Milliseconds).

Condition	**Median**	**Upper quartile**	**Lower quartile**
Full cohort	12696.70	15518.88	9669.96
Resveratrol	13247.49	16356.75	9544.08
Placebo	12178.96	14124.33	9700.23

Analysis of change-from-baseline data observed a significant interaction between treatment*time for ‘percentage correct’ on the name-to-face recall task; *F*(4,380) = 2.49, *p* = 0.04 (Cohens *D*; 0.71). *Post-hoc* exploration of treatment differences at each of the post-dose time-points revealed two significant effects. These effects were at time-points 1 and 2, which relate to the two post-dose assessments during visit 1. In both of these effects, both treatments performed less well compared to baseline on visit 1. However, resveratrol showed a significantly more pronounced reduction at both post-dose repetition 1 [−10.55 (SE; 2.39)], compared to placebo [−1.83 (SE; 2.50)]; *F*(1,96) = 6.32, *p* = 0.01 (Cohens *D*; 0.06), and at post-dose repetition 2 [−12.77 (SE; 2.28)], compared to placebo [−4.00 (SE; 2.52)]; *F*(1,96) = 6.60, *p* = 0.01 (Cohens *D*; 0.07), respectively. See Figure 11B, for Name-to-Face Recall ‘Percentage Correct’ graph.

- Name-to-face recall ‘percentage correct surname’

Analysis of change-from-baseline data observed a significant interaction between treatment*time for ‘percentage correct surname’ on the name-to-face recall task; *F*(4,380) = 2.71, *p* = 0.03 (Cohens *D*; 0.75). The single significant effect here is at time-point 2, relating to the second post-dose assessment during visit 1 and, as above with the overall ‘Percentage Correct’ effect, this was due to participants in the resveratrol condition detecting fewer surnames [−13.83 (SE; 2.90)], than those in the placebo condition [−3.67 (SE; 2.57)]; *F*(1,96) = 6.92, *p* = 0.01 (Cohens *D*; 0.07). See Figure 11C, for Name-to-Face Recall ‘Percentage Correct Surname’ graph.

- Numeric working memory ‘percentage correct’

A significant baseline difference between treatments at visit 2 was observed on the ‘Percentage Correct’ outcome measure of the numeric working memory task. Here, resveratrol performed highest, with a mean of 96.58% (SE; 0.57), compared to the placebo mean of 92.88% (SE; 1.02); *t*(97) = −3.11, *p* = <0.01 (Cohens *D*; −0.63).

Analysis of change-from-baseline data, comparing treatment differences at each of the 5 time-points, revealed a significant interaction between treatment*time; *F*(4,388) = 5.17, *p* = <0.01 (Cohens *D*; 0.97). Exploratory post-hoc comparisons at each time-point revealed this effect to be at time point 3 (i.e., the pre-dose assessment during visit 2), with resveratrol reaching a higher change-from-baseline percentage correct [+3.43% (SE; 1.81)], compared to placebo [−1.13% (SE; 0.82)]; *t*(97) = −0.23, *p* = 0.02 (Cohens *D*; *−*0.47).

**Figure 12:**
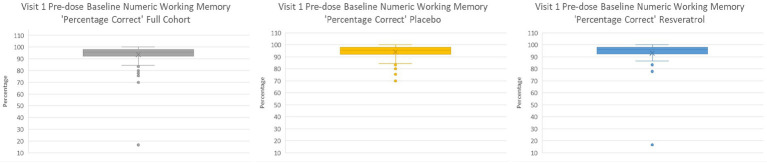
Box and whisker plot observations on visit 1, pre-dose baseline data for numeric working memory ‘percentage correct’.

**Figure 13:**
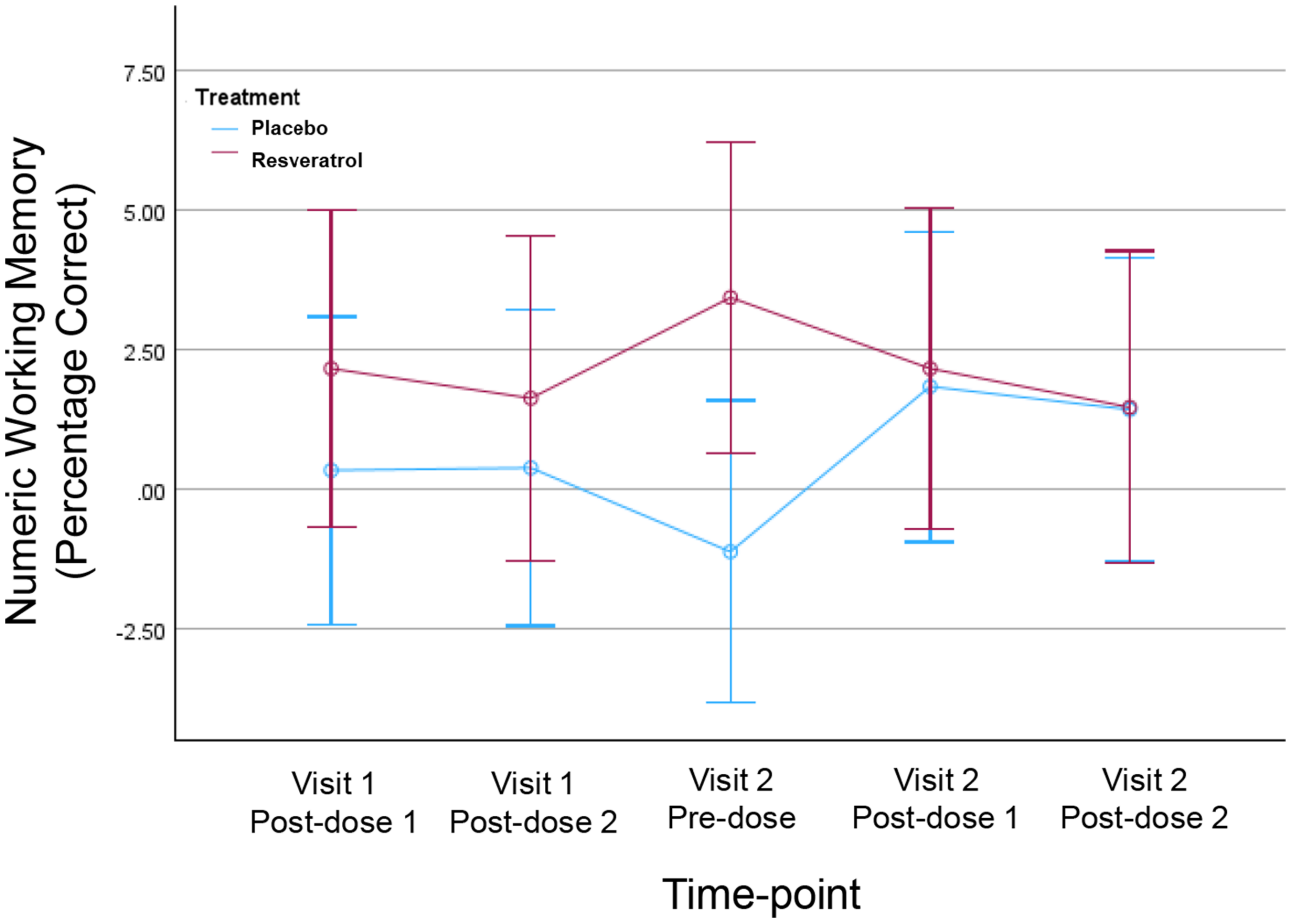
Numeric working memory ‘percentage correct’ change-from-baseline results.

Whilst there was no statistically significant difference between treatment groups at the visit 1, pre-dose baseline time-point, reference to the box and whisker plot information from this time-point does reveal that a salient outlier in the resveratrol condition, performing at approximately 20%, may be skewing interpretation of results here (see Figure 12), which should be considered when interpreting this treatment*time interaction. It’s important to note that this level of performance is still within the normal range of performance for this task, and so there is no justification to remove this datapoint from the analysis. See Figure 13 for Numeric Working Memory ‘Percentage Correct’ graph.

### Gut microbiota

DNA sequencing and rarefaction analysis data denoted that depth of sequencing was sufficient for robust analysis. The analysis determined that 1.0 × 104 reads were needed to reach asymptote. A mean of 3.5 × 104 read depth was taken forward for these analyses.

Comparing overall diversity between participants in both treatment groups, the alpha diversity illustrated that there was no statistical change to the bacterial community diversity within either Amplicon Sequence Variant (*p* = 0.48) and Shannon Diversity (*p*= > 1).

Comparing Beta diversity, the Principal Coordinates Analysis (PCA) found one baseline difference between treatment groups; with a greater abundance of Acidimicrobiia (C111) observed in those receiving resveratrol, compared with the placebo group. No other pre-dose differences were observed, suggesting that the treatment groups were fairly similar in their gut microbial profile before commencing the intervention.

With regards analysis of treatment effects, no statistical difference based on any test variable was observed (study arm *p* = 0.71, participant *p* = 0.074, or individual sample *p* = 1). Interestingly, whilst the study arm (i.e., treatment group) did not find differences in community dissimilarity, there was a high degree of dissimilarity within the placebo condition, with the largest difference based on the individual sampled. Two differentially abundant taxa were observed here; specifically, a reduction in Actinobacteria (ACK M1) and an increase in Clostridia (Ruminococcaceae). No differentially abundant taxa were observed following intervention with resveratrol. One differentially abundant taxa was observed between treatment groups following intervention, with more Bacteroidia (Barnesiellaceae) observed following resveratrol supplementation, when compared with placebo.

### Urinary metabolome

Following LC–MS analysis, positive analysis identified 4,662 mass spectral features. The resulting peak table was sequentially filtered to only include reproducible and stable peaks, which resulted in a final 2,827 features in positive ionization mode. PLS-DA analysis identified 15 mass spectral features which significantly differed between treatment groups. Of these, six were matched to existing metabolite databases (KEGG), and therefore could be identified as Trienoic acid, Sulfonic acid, Dihydroxy-oxo sulfanylium, Dihydroresveratrol 4′ sulfate, Trans-Resveratrol 3,4′ disulfate, and Oxidanesulfonic acid.

Following this, negative analysis identified 3,000 mass spectral features. The resulting peak table was sequentially filtered to only include reproducible and stable peaks, which resulted in a final 1,212 features in negative ionization mode. PLS-DA analysis identified 11 mass spectral features which significantly differed between treatment groups. Of these, five were matched to existing metabolite databases and therefore could be identified as Oxidanesulfonic acid, Dihydroresveratrol 4′ sulfate, Dihydroxy-oxo sulfanylium, Sulfonic acid, and Trans-Resveratrol 3,4′ disulfate, which were all observed in the positive ionization mode above. A table outlining the full mass spectral features of these compounds can be found in the Supplementary materials.

### Blood biomarkers

No significant treatment effects were observed on any of the blood biomarker analyses.

Supporting results (including all outcome measure box and whisker plots, results, adverse events, and compliance) can be found within the Supplementary materials.

## Discussion

To summarize the results, it is probably clearest to separate those which could be indicative of true treatment effects, from those which are more likely to represent naturally superior performance inherent in the placebo condition participants (i.e., those coupled with significant pre-dose effects at visit 1, before the intervention commenced).

Covering these first, we observed five significant effects (out of a total of nine) where preexisting baseline differences stymie interpretation. The first of these, a significant treatment*time interaction for a lower false alarm rate on the Rapid Visual Information Processing task, in the placebo condition, was predicated on a significant pre-dose baseline difference between the treatment groups. As such, further analyses to explore this interaction included ‘quartile group’, and here the treatment effect disappeared, to be replaced by a significant effect of quartile group. This supports the argument that the pre-existing performance level of participants, whether they were in the high, low, or middle quartile, significantly impacted their subsequent ability.

This was the rationale for deviating from the pre-planned statistical approach, choosing instead to consider participants as fundamentally different from each other (both within and between their treatment group) from the start, and throughout the trial, by categorizing them via quartile group. Other analytical approaches, such as considering these differences continuously (i.e., covarying the baseline), did not adequately address this, but incorporating the categorical factor of quartile group had the effect of erasing what we believed to be type I errors. Part of the reason for this was the lack of evidence that resveratrol, at 500 mg, would result in any kind of deficits at all, and certainly not cognitive deficits (28), and so these indications from the original analysis were surprising. As Tu et al. summarize, 500 mg resveratrol is considered an optimal dose for many outcomes, including cognitive improvements in certain models, hence its selection in this study. The compliance measures indicate that our participants were consuming this daily dose, with the returned capsule count showing 76–116% compliance, and so we too were expective positive effects on cognitive function. However, the poor bioavailability of resveratrol is well known (29), and it may be the case that even the chronic supplementation regimen used in this study was insufficient to achieve circulating levels capable of exerting positive cognitive outcomes. Whilst we did attempt to verify this by measuring plasma levels of resveratrol, discussions below explain why this was not possible.

The other four tasks with significant effects of treatment were Serial 3 Subtraction ‘Errors’, Serial 7 Subtraction ‘Total’, Name-to-Face Recall ‘Reaction Time’, and Numeric Working Memory ‘Percentage Correct’. All of these significant treatment effects were limited to pre-dose effects during visit 2, and demonstrated significantly poorer performance in the resveratrol condition, which could initially be interpreted as a negative effect of 12-weeks’ resveratrol supplementation. None of these outcomes were accompanied by a significant pre-dose baseline difference during visit 1, suggesting that a preexisting difference wasn’t detected in the same way that it was for the Rapid Visual Information Processing task above. However, when scrutinizing the box and whisker plots for these outcome measures, stark differences in median and quartile ranges, and outliers, were observed, and these were in the same direction as the abovementioned Rapid Visual Information Processing task; i.e. with better natural performance in the placebo condition. It seems axiomatic that this naturally superior ability in the placebo condition explains all five of these effects, even though the latter were not detected statistically, and that what appear to be detrimental effects in response to resveratrol, are in fact just null effects in all cases.

The occurrence of pre-existing effects is very difficult to protect against in randomized controlled trials. For context, all participants underwent a training/screening visit prior to enrolment into this trial, where health and lifestyle factors were recorded, and the cognitive tasks were practiced. This session lasted approximately 2–3 h and, during this time, the cognitive tasks were practiced several times. In order to progress to enrolment, participants were required to meet predetermined thresholds on all of these tasks (calculated by aggregating almost two decades of performance data on these tasks within our own lab), to ensure a level of understanding that was equal amongst the entire cohort. As such, we are able to ensure that a lower threshold is met by all participants. However, upper thresholds were not set, and so there can exist a wide variance in cognitive ability amongst enrolled participants. It is then possible for a greater proportion of ‘higher performers’ to be randomly assigned to one treatment condition above another, which we believe is the case here with regards the placebo condition. In an attempt to protect against this, future trials could consider categorizing participants based on their pre-enrolment aptitude, and then factoring this into the randomization schedule. However, this would have to be weighed against constraints such as natural variability in cognitive performance, which might mask true ability at baseline, and the opposite scenario, where participants appear to cease effort on the cognitive tasks during the trial itself; appearing as a reduction in ability from baseline. This is a separate discussion point however, and will be discussed further below.

A secondary issue with the abovementioned baseline differences is that, whilst they were detected statistically on Rapid Visual Information Processing ‘False Alarms’, and additionally interpreted from the box and whisker plot information for Serial 3 Subtraction ‘Errors’, Serial 7 Subtraction ‘Total’, Name-to-Face Recall ‘Reaction Time’, and Numeric Working Memory ‘Percentage Correct’, this could represent an overall naturally superior cognitive ability which has impacted other tasks and outcome measures. Specifically, this would pertain to the remaining four of the nine outcome measures where significant effects were observed; Name-to-Face Recall ‘Percentage Correct’, Name-to-Face Recall ‘Percentage Correct Surname’, Profile Of Mood States ‘Anger/Hostility’, and Heart Rate. However, it’s important to note that these outcomes were free of interpretable baseline differences between treatment groups, and could be interpreted as true negative performance outcomes in the resveratrol condition.

Moving on to other study outcome measures, we observed the presence of several urinary biomarkers in resveratrol supplemented participants. These comprised Trienoic acid, Sulfonic acid, Dihydroxy-oxo sulfanylium, Dihydroresveratrol 4′ sulfate, Trans-Resveratrol 3,4′ disulfate, and Oxidanesulfonic acid. None of these compounds are particularly noteworthy, aside from the presence of Trans-Resveratrol 3,4′ disulfate, which indicates that the ingested parent compound had been converted to this conjugate prior to urinary excretion. However, the others are all fairly rudimentary compounds, with no salient bioactive effects, and so no further interpretation is not warranted.

What is interesting, given the urinary detection of Trans-Resveratrol 3,4′ disulfate, is that no resveratrol markers, parent or conjugates, were observed in the blood biomarker analyses, nor were any other significant differences in the blood biomarkers observed (comprising cholesterol, low-density lipoprotein (LDL), high-density lipoprotein (HDL), triglycerides, C-reactive protein (CRP), interleukin-6 (IL-6), ferric reducing antioxidant power (FRAP), glucose, resveratrol, resveratrol-3-O-D-glucoside, resveratrol-3-0-sulfate, and resveratrol-4-O-D-glucoronide). The likely answer to this is that we simply had insufficient samples to power these analyses. Sample sizes for full datasets here (i.e., a pre- and post-dose sample on visit 1, and a pre- and post-dose sample on visit 2) were incredibly small. IL-6 had insufficient datasets to run any analysis (*N* = 2), and the other markers ranged between *N* = 12 (for FRAP), and *N* = 22 (for resveratrol). Reasons for these low numbers include the inability to obtain a sufficient quantity of intravenous blood from participants to perform the appropriate tests, and the apparent absence of the biomarker from the sample, due to not meeting the threshold for detection. Whilst the current study wasn’t powered to detect an effect on these biological markers, it’s likely that numbers were simply too small to observe differences in these exploratory analyses. Having said this, many pharmacokinetic studies assessing resveratrol bioavailability have observed effects in smaller cohorts; e.g., *N* = 7 (5), so it may be that more factors are influencing the null results, such as heterogeneity within the cohort. Again, these are variables which can be corrected with relative ease in future studies, by ensuring that sample sizes are sufficient and that groups are as homogenous as possible.

The issue of inter-individual variability within the cohort is likely also the reason that no changes were observed in the gut microbiome of participants. A large natural variance amongst the entire cohort was observed at baseline, and this noise can make interpreting any change challenging. Other groups have remedied this by focusing on groups with a more restricted and homogenous diet, such as athletes, and report this to be a more sensitive baseline to dietary intervention-induced microbiome changes (30). For studies where such microbiome assessments are the primary outcome measure, this niche participant pool is logical. However, this may not be possible for assessing outcomes like cognitive function, as was the case here, where results need to be generalized to a wider population. An important consideration to raise here is that, whist the resveratrol microbiome research that inspired the rationale here did observe changes in the gut microbiome, these trials were *in vitro* (18), and in rodent models (19), where the relative simplicity of the models may be more conducive to change. It’s also a reasonable assumption that the participants in the current trial presented with what could be defined as a healthy microbiome, given their general good health and wellbeing, and that no clinical microbial profiles were identified at baseline, and so the fact that the investigational product did not induce shifts within this environment can be regarded as a positive one. An interesting future avenue would be to investigate participants presenting with defined gut dysbiosis, to assess whether a gut environment in need of positive shifts is more malleable to change. As a result, this model may additionally be more conducive to augmentation of cognition and inflammation.

### Strengths and limitations

The key strength of this trial is in the well-powered cognitive analysis of resveratrol supplementation within a randomized controlled trial. The *A priori* power calculation determined that *N* = 102 was required to sufficiently power the cognitive primary outcome measure, and *N* = 99 was achieved, a number which is rare in dietary intervention trials, especially with resveratrol. However, it is noteworthy that some of the individual cognitive tasks were less well-powered than this, due to the need to remove a significant number of participant data. This was particularly salient for the Serial 3 and Serial 7 Subtraction, and Rapid Visual Information Processing tasks, where *N* = 5, *N* = 8, and *N* = 14, respectively, data sets had to be removed prior to analysis for non-completion/engagement with the tasks by participants. This resulted in *N* = 94, *N* = 91, and *N* = 85 datasets for these analyses, respectively (all other outcome measures were higher than this), which are not adequately powered.

This lack of completion/engagement of tasks by participants was determined by interrogating visit 1 and visit 2 data, pre-un-blinding, for scores which fell below the threshold for understanding the tasks, and highlighting those who consistently fell below these thresholds. This is an issue which has pervaded previous research within our lab, and an issue which is now being countered by alerting participants to the fact that data is being monitored within the intervention period itself, as well as the screening/training session. The data in question is unpublished, but anecdotally this has made a huge difference to preventing data loss, suggesting that it was indeed an issue with lack of engagement, and should be considered for future research.

A related weakness of the trial is the amount of data which was lost from the blood biomarker analyses, which was so low in the case of IL-6 that this analysis could not be performed. As part of the issue was the inability to obtain four samples, of sufficient quantity, from the desired number of participants, future trials should arguably hone in on fewer biomarkers for assessment, requiring a smaller quantity of blood, and recruit participants who are sufficiently physically robust to provide these samples; as we have done previously by recruiting a sub-sample of male participants who were all physically active (4). Secondly, a large proportion of biomarkers simply did not meet the threshold for detection according to the analytical techniques applied, and so it may be that these approaches need to be re-thought for future trials.

## Conclusion

No significant effects were observed under the conditions of this study. The heterogeneity of the study population, the existing healthy microbial and cognitive status of participants, as well as the limited sample sizes in the biological marker analyses, likely impact the results here. The effects reported in the placebo control group are argued to represent the persistence of preexisting differences in cognitive ability established at the pre-dose baseline, a situation which makes interpretation of findings across this study very challenging. The lack of change to a presumed healthy microbiome in resveratrol-supplemented participants can be regarded as positive, but future research may want to consider identifying cohorts with defined gut dysbiosis, to ascertain whether resveratrol supplementation can improve an unhealthy microbiome through prebiotic actions. Finally, given the relatively high performance of the cohort within this study, it’s worth considering whether high body mass index represents an effective model of compromised cognition. Other groups have observed more impact in different models, such as post-menopause (31), although a recent meta-analysis has questioned the robustness of this (32); observing, as we did, no concomitant change in the metabolic parameters which would underpin cognition. As such, should future research investigate women at earlier key hormonal life-stages, such as peri-menopause, before these metabolic parameters become resistant to change?

## Data Availability

The raw data supporting the conclusions of this article will be made available by the authors, without undue reservation.
