# Supplementary material for: The effects of 12-weeks resveratrol supplementation on cognition, gastrointestinal microbiota, and systemic inflammation, in an overweight and obese human population: a randomized, double-blind, placebo controlled, parallel groups trial
Source: Front Nutr. 2026 Jul 1;13:1839709. doi: 10.3389/fnut.2026.1839709 (PMC13371205; doi:10.3389/fnut.2026.1839709)
Supplement: Supplementary file 1 [file Table_1.DOCX]

**Contents**

[**1. Full Exclusion Criteria List** 3](#_Toc225409487)

[**2. Meal composition** 4](#_Toc225409488)

[**3. Adverse Events** 5](#_Toc225409489)

[**4. Compliance** 6](#_Toc225409490)

[**5. Box and Whisker Plots for Nine Significant Outcome Measures** 7](#_Toc225409491)

[**6. Table 1. Identified Mass Spectral Features.** 9](#_Toc225409492)

[**7. All Results** 10](#_Toc225409493)

[**7.1. Blood Biomarkers** 10](#_Toc225409494)

[7.1.1 Cholesterol 10](#_Toc225409495)

[7.1.2 High Density Lipoprotein (HDL) 10](#_Toc225409496)

[7.1.3 Low Density Lipoprotein (LDL) 10](#_Toc225409497)

[7.1.4 Triglycerides 11](#_Toc225409498)

[7.1.5 Glucose 11](#_Toc225409499)

[7.1.6 C-reactive protein (CRP) 11](#_Toc225409500)

[7.1.7 Ferric Reducing Antioxidant Power (FRAP) 11](#_Toc225409501)

[7.1.8 Resveratrol 11](#_Toc225409502)

[7.1.9 Resveratrol-3-O-Sulphate 12](#_Toc225409503)

[7.1.10 Resveratrol-3-Glucoronide 12](#_Toc225409504)

[7.1.11 Resveratrol-4-Glucoronide 12](#_Toc225409505)

[**7.2. Body Mass Index** 12](#_Toc225409506)

[**7.3. Blood Pressure** 13](#_Toc225409507)

[7.3.1 Systolic 13](#_Toc225409508)

[7.3.2 Diastolic 13](#_Toc225409509)

[7.3.3 Heart Rate 13](#_Toc225409510)

[**7.4. COMPASS Tasks** 13](#_Toc225409511)

[7.4.1 Serial 3 Subtractions 13](#_Toc225409512)

[7.4.2 Serial 7 Subtractions 16](#_Toc225409513)

[7.4.3 Rapid Visual Information Processing 19](#_Toc225409514)

[7.4.4 Word Recall 22](#_Toc225409515)

[7.4.5 Name to Face Recall 23](#_Toc225409516)

[7.4.6 Numeric Working Memory 25](#_Toc225409517)

[7.4.7 Choice Reaction Time 26](#_Toc225409518)

[7.4.8 Corsi Blocks (Span Score) 27](#_Toc225409519)

[7.4.9 Peg and Ball 27](#_Toc225409520)

[7.4.10 Picture Recognition 28](#_Toc225409521)

[7.4.11 Word Recognition 29](#_Toc225409522)

[**7.5. Mood** 29](#_Toc225409523)

[7.5.1 Profile Of Mood States (POMS) 29](#_Toc225409524)

[7.5.2 Bond Lader (completed during COMPASS task battery) 31](#_Toc225409525)

[7.5.3 Visual Analogue Scales (completed during COMPASS task battery) 32](#_Toc225409526)

[**7.6. Summary of Significant Effects** 34](#_Toc225409527)

[**7.6.2 Results Summary** 38](#_Toc225409528)

# **1. Full Exclusion Criteria List**

- Failure to meet any one of the inclusion criteria
- Have taken antibiotics (including pre- and pro-biotic supplements/drinks; e.g. Yakult or Actimel) during the previous 8 weeks
- For those providing stool samples, have irregular bowel movements (less than 1 per day)
- Have any pre-existing medical conditions/illness (this was with the exception of controlled hypo/hyperthyroidism, mild asthma, hay fever, reflux-related conditions, and high cholesterol)
- Have type I or type II diabetes
- Currently taking prescription medications (this was with the exception of contraceptives, Hormone Replacement Therapies (HRT), medications used in the treatment of hypo/hyperthyroidism, reflux medication, and as required in the treatment of hay fever and mild asthma)
- Have a visual impairment that cannot be corrected by glasses or contact lenses, including colour blindness
- English not being the participants first language, or proficiency not equivalent to IELTS band 6 or above
- Have any learning difficulties or dyslexia
- Suffer from frequent migraines that require medication (> 1 per month)
- Have any food allergies, intolerances or sensitivities
- Have high blood pressure (systolic over 159 mm Hg or diastolic over 99 mm Hg)
- Smoke tobacco, vape nicotine or use nicotine replacement products
- Have a history of alcohol or drug abuse
- Pregnant, seeking to become pregnant or lactating
- Unable to complete all of the study assessments
- Participating in any other clinical or nutritional intervention trials, or have done within the previous 4 weeks
- Have any health condition that would prevent fulfilment of the study requirements
- Have habitually used supplements within the previous month (defined as more than 3 consecutive days, or 4 days in total)
- Consume large amounts of caffeine (> 500mg per day; equivalent to 5/6 cups of coffee/8 cups of tea per day, or equivalent from other sources)
- Consume more than 5 portions of fruit or vegetables per day
- Any sleep disturbances or take sleep aid medication
- Have any known active infections
- Have/risk of having syphilis, hepatitis, the Human T-lymphotropic virus or the Human Immunodeficiency Virus
- History of breast cancer and/or a mastectomy
- History of haemophilia or any similar clotting disorder
- Do not have a bank account (required for payment)

# **2. Meal composition**

*Standardised breakfast*

2 slices of toasted Hovis soft white bread: 186 kcal, 1.4g fat, 2.8g sugar, 7g protein

Lurpak slightly salted spread 15 g : 106 kcal, 11.7 g fat, <0.1 g sugar, <0.11 g protein

*Optional snack*

Sainsbury’s Gold Roast Decaffeinated Instant Coffee: 1 kcal, 0 g fat, 0 g sugar, 0 g protein

Tetley’s Decaffeinated teabags: 1 kcal, 0 g fat, 0 g sugar, 0 g protein

Semi Skimmed milk (if desired) ~10 ml: 5 kcal, 0.1 g fat, 0.4 g sugar, 0.3 g protein

*Standardised lunch*

Hovis soft white bread x 2 slices: 186 kcal, 1.4 g fat, 2.8 g sugar, 7 g protein

Sainsbury’s British Medium Grated Cheddar Cheese 30 g: 127 kcal, 10.5 g fat, <0.5 g sugar, 7.6 g protein

Lurpak slightly salted spread ~10 g: 72 kcal, 8 g fat, <0.1 g sugar, <0.1 g protein

Walkers Ready Salted Crisps 25 g bag: 132 kcal, 8 g fat, 0.1 g sugar, 1.5 g protein

Ambrosia 125 g pot (due to an ordering error, some participants consumed the light versions (values in italics) for both visits): 124/*113* kcal, 3.5/ *2.3* g fat, 14.3/*13.8* g sugar, 3.6/*3.6* g protein

Total nutritional value of the meal calculated as (Ambrosia light values in italics): 641/*630* kcal, 31.4/*30.2* g fat, 17.8/*17.3* g sugar, 19.8/*19.8* g protein.

# **3. Adverse Events**

Over the course of the study, 138 adverse events (AEs) presented which could possibly be related to the study treatment (75 treatment A; 63 treatment B). These comprised:

• Headache/migraine: 57 individual reports from 28 participants

• Gastrointestinal: 16 individual reports from 12 participants

• Muscular/bodily pain/injury: 12

• Heartburn/indigestion: 16

• Cold/flu symptoms: 17

• Vomiting/nausea/sickness: 2

• Vitamin D deficiency: 1

• Blurred vision: 1

• Haemorrhoids: 1

• Hair loss: 1

• Rash: 3 individual reports from 1 participant

• Urinary tract infection: 1

• Increased feeling of hunger: 1

• Sciatica: 1

• Mouth ulcer: 1

• Cellulitis: 1

• Impetigo: 1

• Change in breast tissue: 1

• Helicobacter pylori: 1

• Thrush (caused by antibiotics): 2 individual cases reported from 1 participant

All symptoms in all cases resolved during the course of the study, with the exception of 4 participants who ceased participation due to AEs (2 gastrointestinal, 1 migraine, and 1 change in breast tissue), but follow up indicated that all issues had resolved and that participants were in good health. AEs were reported as; 95 ‘mild’, 34 ‘moderate’, 2/3 ‘moderate/severe’, and 7 ‘severe’.

Six participants were removed from the analysis due to consumption of antibiotics during the supplementation period, therefore breaching the exclusion criteria. These were consumed by participants reporting shingles, urinary tract infection, ear infection, cellulitis, impetigo and helicobacter pylori.

# **4. Compliance**

The treatment consumption period was intended to last 84 +/-5 days. The actual supplementation period ranged from 76 – 99 days. The primary compliance measure was a capsule count upon return to the lab for visit 2. A secondary measure, completion of a treatment diary noting the time of treatment consumption each day, was utilized to support this information. Mean compliance was 96%, ranging 70-116%. This meant that N=4 was outside of the >80/<120% compliance range set for inclusion in analyses, N=1 (with 70% compliance) was excluded due to antibiotic use, N=1 (with 72% compliance) was excluded for being outside the range, and it was decided to include the remaining N=2 (both with 76% compliance) in the analysis, given how close they were to the desired range.

# **5. Box and Whisker Plots for Nine Significant Outcome Measures**

**6. Table 1. Identified Mass Spectral Features.** Identified from both negative and positive mode that are influenced following resveratrol intervention.

| Feature ID | Mass (m/z) | Formula | Compound name |
| --- | --- | --- | --- |
| Positive mode ionisation analysis | | | |
| M0915 | 180.11.417 | C11 H16 O2 | Trienoic acid |
| M0023 | 340.06.226 | C15 H16 O7S | Sulfonic acid |
| M0011 | 420.01.781 | C18 H12 O10S | Dihydroxy-oxo-sulfanylium |
| M0049 | 310.05.164 | C14 H14 O6S | Dihydroresveratrol 4'-sulfate |
| M0015 | 387.99121 | C14 H12 09 S2 | Trans-Resveratrol 3,4'-disulfate |
| M0164 | 516.09.431 | C21 H24 O13S | Oxidanesulfonic acid |
| Negative mode ionisation analysis | | | |
| M0164 | 516.09.431 | C21 H24 O13S | Oxidanesulfonic acid |
| M0049 | 310.05.164 | C14 H14 O6S | Dihydroresveratrol 4'-sulfate |
| M0011 | 420.01.781 | C18 H12 O10S | Dihydroxy-oxo-sulfanylium |
| M0023 | 340.06.226 | C15 H16 O7S | Sulfonic acid |
| M0015 | 387.99121 | C14 H12 09 S2 | Trans-Resveratrol 3,4'-disulfate |

# **7. All Results**

## **7.1. Blood Biomarkers**

Independent samples t tests were performed on the baseline samples from each of the 2 visits, with ‘Treatment’ as the grouping variable, to determine the presence of any true baseline differences between participants (i.e. comparing treatment effects on visit 1 baseline), and pure chronic effects of treatment (i.e. comparing treatment effects on visit 2 baseline).

To compare the main effects of treatment over time, the post-dose sample on day 1, the pre-dose sample on day 2, and the post-dose sample on day 2 were converted to change-from-day 1-baseline. A repeated measures ANOVA was then performed with ‘Time’ as the within-subjects factor (with 3 levels; post-dose visit 1, pre-dose visit 2, and post-dose visit 2), and ‘Treatment’ as the between subjects factor. Bonferroni corrections were applied.

The T and F tests are reported below, alongside observed power (*Cohens D*) in brackets. Only a significant main effect of treatment, or a significant interaction between treatment*time, will be explored further with post-hoc analyses (i.e. a main effect of time will not be further explored).

### 7.1.1 Cholesterol

Those in the placebo condition began the study with significantly higher cholesterol (mean; 203.52 mmol/L, SD; 44.88) than those in resveratrol (mean; 140.34 mmol/L, SD; 13.88); t(15)=4.03, p=<.01 (*1.96*). There were no effects of treatment on the visit 2 baseline data; t(15)=-.04, p=.97. (*-.02*).

The repeated measures analysis revealed no significant effect of time; F(2,30)= .91, p=.41 (*.19*), nor a treatment*time interaction; F(2,30)= 1.35, p=.27 (*.27*). A main effect of treatment was observed; F(1,15)= 5.10, p=.04 (*.56*), with those in placebo showing the greatest reduction in cholesterol levels overall (mean; -25.24, SE; 15.67), compared to resveratrol (mean; 23.39, SE; 14.78). However, this is not surprising given that those in placebo began the trial with significantly higher levels of cholesterol.

### 7.1.2 High Density Lipoprotein (HDL)

There were no significant differences between treatment groups at visit 1 baseline; t(13)= 1.02, p=.33 (*.53*), nor visit 2 baseline; t(13)= .79, p=.45 (*.41*).

The repeated measures analysis revealed no significant effect of time; F(2,26)= 1.82, p=.18 (*.35*), no interaction between treatment*time; F(2,26)= .32, p=.73 (*.10*), nor a main effect of treatment; F(1,13)= .02, p=.90 (*.05*).

### 7.1.3 Low Density Lipoprotein (LDL)

There were no significant differences between treatment groups at visit 1 baseline; t(12)= -1.23, p=.24 (*-.66*), nor visit 2 baseline; t(12)= -1.38, p=.19 (*-.74*).

The repeated measures analysis revealed a significant effect of time; F(2,24)= 88.18, p=<.01 (*1.00*), but no significant interaction between treatment*time; F(2,24)= .27, p=.77 (*.09*), nor main effect of treatment; F(1,12)= .03, p=.87 (*.05*).

### 7.1.4 Triglycerides

There were no significant differences between treatment groups at visit 1 baseline; t(13)= -.20, p=.85 (*-.10*), nor visit 2 baseline; t(13)=.31, p=.76 (*-.16*).

The repeated measures analysis revealed a significant effect of time; F(2,26)= 4.02, p=.03 (*.67*), but no significant interaction between treatment*time; F(2,26)= 1.23, p=.31 (*.24*), nor main effect of treatment; F(1,13)= .57, p=.47 (*.11*).

### 7.1.5 Glucose

There were no significant differences between treatment groups at visit 1 baseline; t(13)= .24, p=.81 (*-.13*), nor at visit 2 baseline; t(13)= -.55, p=.59 (*-.29*).

The repeated measures analysis revealed no significant main effect of time; F(2,26)= .70, p=.51 (*.16*), no significant interaction between treatment*time; F(2,26)= 1.53, p=.24 (*.30*), and no main effect of treatment; F(1,13)= .47, p=.51 (*.10*).

### 7.1.6 C-reactive protein (CRP)

There were no significant differences between treatment groups at visit 1 baseline; t(13)=.81, p=.43 (*.42*), nor at visit 2 baseline; t(13)= .40, p=.70 (*.21*).

The repeated measures analysis revealed a significant main effect of time; F(2,26)= 8.44, p=<.01 (*.94*), but no significant interaction between treatment*time; F(2,26)= 1.64, p=.21 (*.31*), and no significant main effect of treatment; F(1,13)= .26, p=.62 (*.08*).

### 7.1.7 Ferric Reducing Antioxidant Power (FRAP)

There were no significant differences between treatment groups at visit 1 baseline; t(10)= .34, p=.74 (*.20*), nor at visit 2 baseline; t(10)= 1.14, p=.28 (*.67*).

The repeated measures analysis revealed no significant main effect of time; F(2,20)= .22, p=.81 (*.08*), no significant interaction between treatment*time; F(2,20)= 1.32, p=.29 (*.25*), nor significant main effect of treatment; F(1,10)= .01, p=.94 (*.05*).

### 7.1.8 Resveratrol

There were no significant differences between treatment groups at visit 1 baseline; t(20)= -.12, p=.91 (*-.05*), nor at visit 2 baseline; t(20)= .69, p=.50 (*.29*).

The repeated measures analyses revealed a trend towards a significant main effect of time: F(2,40)= 3.12, p=.06 (*.57*), but no significant interaction between treatment*time; F(2,40)= .44, p=.65 (*.12*), nor a significant main effect of treatment; F(1,20)= .02, p=.89 (*.05*).

### 7.1.9 Resveratrol-3-O-Sulphate

There were no significant differences between treatment groups at visit 1 baseline; t(12)= -.08, p=.94 (*-.04*), nor at visit 2 baseline (*.16*).

The repeated measures analysis revealed no significant main effect of time; F(2,24)= .52, p=.60 (*.13*), no significant interaction between treatment*time; F(2,24)= .57, p=.58 (*.13*), and no significant main effect of treatment; F(1,12)= .90, p=.36 (*.14*).

### 7.1.10 Resveratrol-3-Glucoronide

There were no significant differences between treatment groups at visit 1 baseline; t(16)= -.71, p=.49 (*-.34*), nor at visit 2 baseline; t(16)= -.80, p=.43 (*-.38*).

The repeated measures analysis revealed no significant main effect of time; F(2,32)= .36, p=.70 (*.10*), no significant interaction between treatment*time; F(2,32)= .74, p=.49 (*.16*), and no significant main effect of treatment; F(1,16)= .45, p=.51 (*.10*).

### 7.1.11 Resveratrol-4-Glucoronide

There were no significant differences between treatment groups at visit 1 baseline; t(16)= .07, p=.94 (*.04*), nor at visit 2 baseline; t(16)= .52, p=.61 (*.25*).

The repeated measures analysis revealed no significant main effect of time; F(2,32)= .29, p=.75 (*.09*), no significant interaction between treatment*time; F(2,32)= .19, p=.83 (*.08*), and no significant main effect of treatment; F(1,16)= .02, p=.89 (*.05*).

## **7.2. Body Mass Index**

An independent samples t tests was performed on the visit 1 measurement of Body Mass Index, with ‘Treatment’ as the grouping variable, to determine the presence of any true baseline differences between participants.

To compare the main effects of treatment over time, the visit 2 measurement of Body Mass Index was converted to change-from-baseline, and an independent samples t test compared the effects of treatment here.

There were no significant differences between treatment groups at visit 1 baseline; t(97)= -.16, p=.87 (*-.03*), and no significant effects of treatment on the visit 2 change-from-baseline Body Mass Index; t(97)= .89, p=.38 (*.18*).

## **7.3. Blood Pressure**

Analysis was as per the Blood Biomarkers above. The exception is that the factor of time has 5 levels for Blood Pressure, as readings were taken at 6 points over the course of the study (3 per visit), creating 5 change-from-baseline measures.

### 7.3.1 Systolic

There were no significant differences between treatment groups at visit 1 baseline; t(96)= -.03, p=.98 (*-.01*), nor at visit 2 baseline; t(96)= -.23, p=.82 (*-.05*).

The repeated measures analysis revealed no significant main effect of time; F(4,384)= .81, p=.52 (*.26*), no significant interaction between treatment*time; F(4,384)= 1.39, p=.24 (*.43*), and no significant main effect of treatment; F(1,96)= .86, p=.36 (*.15*)

### 7.3.2 Diastolic

There were no significant differences between treatment groups at visit 1 baseline; t(96)= .22, p=.83 (*.05*), nor at visit 2 baseline; t(96)= -.94, p=.35 (*-.19*).

The repeated measures analysis revealed a significant trend towards significance for a main effect of time; F(4,384)= 2.10, p=.08 (*.62*), but no treatment*time interaction; F(4,384)= .40, p=.81 (*.14*), and no significant main effect of treatment; F(1,96)= 2.74, p=.10 (*.37*).

### 7.3.3 Heart Rate

There were no significant differences between treatment groups at visit 1 baseline; t(96)= 1.25, p=.21 (*.25*), nor at visit 2 baseline; t(96)= .01, p=.99 (*.002*).

The repeated measures analysis revealed a significant main effect of time; F(4,384)= 62.11, p=<.01 (*1.00*), no interaction between treatment*time; F(4,384)= .24, p=.91 (*.10*), but a significant main effect of treatment was observed; F(1,96)= 7.88, p=<.05 (*.79*). Here, heart rate was significantly lower overall, relative to visit 1 baseline, in the placebo condition (mean change; -1.37 beats-per-minute (SE; .72)), compared to resveratrol (mean change; 1.55 beats-per-minute (SE; .75)).

## **7.4. COMPASS Tasks**

Analysis was as per the Blood Biomarkers and Blood Pressure above. The exception is that the factor of time has 15 levels for the Serial 3 Subtractions, Serial 7 Subtractions, and Rapid Visual Information Processing tasks, as these were repeated 3 times at each of the 3 assessments each day (i.e. 18 completions across the course of the study), creating 15 change-from-baseline measures.

### 7.4.1 Serial 3 Subtractions

#### 7.4.1.1 Total

Baseline differences at visit 1:

Repetition 1: t(92)= 1.90, p=.06 (*.39*)

Repetition 2: t(92)= 1.78, p=.08 (*.37*)

Repetition 3: t(92)= 2.19, p=.03 (*.45*)

Baseline differences at visit 2:

Repetition 1: t(92)= 2.05, p=.04 (*.42*)

Repetition 2: t(92)= 2.39, p=.02 (*.49*)

Repetition 3: t(92)= 2.44, p=.02 (*.50*)

The independent samples t tests revealed significant differences between treatment at baseline, with placebo outperforming resveratrol on all 3 repetitions at visit 2.

Repetition 1 means and standard errors: 36.15 (2.08) versus 30.55 (1.78)

Repetition 2 means and standard errors: 39.47 (2.07) versus 32.96 (1.78)

Repetition 3 means and standard errors: 40.57 (2.03) versus 34.11 (1.70)

However, these baseline differences were present at visit 1 baseline, with 2 trends towards significance (at repetitions 1 and 2), and one significant effect (at repetition 3; with placebo participants averaging 37.45 (1.91) subtractions, versus 31.85 (1.69) for resveratrol). Taken together, this effect looks like a true baseline difference between treatment groups, which has persisted into visit 2. This is supported by the lack of any treatment effects on the repeated measures ANOVA.

The results of the repeated measures ANOVA revealed a significant effect of time; F(14,1288)= 11.25, p=<.01 (*1.00*), but no significant interaction between treatment*time; F(14,1288)= .97, p=.48 (*.63*), nor a significant effect of treatment; F(1,92)= 1.44, p=.23 (*.22*).

#### 7.4.1.2 Correct

Baseline differences at visit 1:

Repetition 1: t(92)= 1.71, p=.09 (*.35*)

Repetition 2: t(92)= 1.88, p=.06 (*.39*)

Repetition 3: t(92)= 2.14, p=.04 (*.44*)

Baseline differences at visit 2:

Repetition 1: t(92)= 2.37, p=.02 (*.49*)

Repetition 2: t(92)= 2.59, p=.01 (*.53*)

Repetition 3: t(92)= 2.52, p=.01 (*.52*)

The same effect was observed on baseline differences here, as per Serial 3s Total. Significant treatment differences were observed on all 3 repetitions at visit 2, with those consuming placebo always performing more correct subtractions than those consuming resveratrol:

Repetition 1 means and standard errors: 34.85 (2.11) versus 28.34 (1.75)

Repetition 2 means and standard errors: 37.66 (2.14) versus 30.26 (1.89)

Repetition 3 means and standard errors: 38.68 (2.10) versus 31.79 (1.76)

However, this was mirrored with a significant effect at repetition 3 during visit 1 (35.34 (2.03) for placebo, and 29.49 (1.82) for resveratrol), which is again suggestive of a true difference between participants on this outcome measure.

The results of the repeated measures ANOVA revealed a significant effect of time; F(14,1288)= 6.94, p=<.01 (*1.00*), but no significant interaction between treatment*time; F(14,1288)= .73, p=.75 (*.48*), and no significant effect of treatment; F(1,92)= 2.21, p=.14 (*.31*).

#### 7.4.1.3 Errors

Baseline differences at visit 1:

Repetition 1: t(92)= 1.11, p=.27 (*.23*)

Repetition 2: t(92)= -.77, p=.45 (*-.16*)

Repetition 3: t(92)= -.43, p=.67 (*-.09*)

Baseline differences at visit 2:

Repetition 1: t(92)= -2.17, p=.03 (*-.45*)

Repetition 2: t(92)= -1.91, p=.06 (*-.39*)

Repetition 3: t(92)= -.97, p=.34 (*-.20*)

A single significant baseline difference on Serial 3 subtraction errors was observed at repetition 1 during visit 2. Here, participants consuming placebo made approximately 1 fewer errors (1.30 (.25)), compared to those consuming resveratrol (2.21 (.34)). However, in the absence of any other significant effects (although repetition 2 was trending towards significance at .06) at pre- or post-dose on visit 2, this effect should be treated with caution.

The results of the repeated measures ANOVA revealed no significant main effect of time; F(14,1288)= 1.03, p=.42 (*.66*), no significant interaction between treatment*time; F(14, 1288)= .79, p=.68 (*.52*), and no significant main effect of treatment; F(1,92)= 1.79, p=.18 (*.26*).

### 7.4.2 Serial 7 Subtractions

#### 7.4.2.1 Total

Baseline differences at visit 1:

Repetition 1: t(89)= 1.75, p=.09 (*.37*)

Repetition 2: t(89)= 1.90, p=.06 (*.40*)

Repetition 3: t(89)= 1.19, p=.24 (*.25*)

Baseline differences at visit 2:

Repetition 1: t(89)= 1.73, p=.09 (*.36*)

Repetition 2: t(89)= 1.52, p=.13 (*.32*)

Repetition 3: t(89)= 2.06, p=.04 (*.43*)

A significant baseline difference was observed on the 3^rd^, and final, baseline repetition on visit 2. Here, participants in the placebo condition completed significantly more subtractions (26.84 (1.56)) than those consuming resveratrol (22.31 (1.53)). This represents a chronic effect of treatment.

The results of the repeated measures ANOVA revealed a significant effect of time; F(14,1246)= 3.87, p=<.01 (*1.00*), a trend towards a significant interaction between treatment*time; F(14,1246)= 2.55, p=<.05 (*.99*), but no significant main effect of treatment; F(1,89)= 1.13, p=.29 (*.18*).

To investigate this trending significant interaction between treatment*time, a one-way ANOVA compared the change-from-baseline performance of those in the placebo condition, to those in resveratrol, at all 15 time-points:

1. F(1,90)= .14, p=.71 (*.00*)

2. F(1,90)= .46, p=.50 (*.01*)

3. F(1,90)= 3.22, p=.08 (*.04*)

4. F(1,90)= 1.97, p=.16 (*.02*)

5. F(1,90)= 2.30, p=.13 (*.03*)

6. F(1,90)= .02, p=.89 (*.00*)

7. F(1,90)= .25, p=.62 (*.00*)

8. F(1,90)= .33, p=.57 (*.00*)

9. F(1,90)= 4.55, p=.04 (*.05*)

10. F(1,90)= .73, p=.39 (*.01*)

11. F(1,90)= 3.41, p=.07 (*.04*)

12. F(1,90)= 4.42, p=.04 (*.05*)

13. F(1,90)= 1.06, p=.31 (*.01*)

14. F(1,90)= .21, p=.65 (*.00*)

15. F(1,90)= 5.75, p=.02 (*.06*)

Three significant effects emerged; at time-point 9 (relating to the visit 2 baseline assessment, specifically the 3^rd^ repetition), where those consuming placebo completed significantly more subtractions (2.39 (.61)), compared to resveratrol (.31 (.77)); time-point 12 (relating to the first post-dose assessment during visit 2, specifically the 3^rd^ repetition), where those consuming placebo completed significantly more subtractions (3.02 (.65)) compared to resveratrol (.98 (.73)); and at time-point 15 (relating to the second assessment during visit 2, specifically the 3^rd^ repetition), where those consuming placebo completed significantly more subtractions (2.76 (.62)) compared to resveratrol (.19 (.90)).

#### 7.4.2.2 Correct

Baseline differences at visit 1:

Repetition 1: t(89)= 1.68, p=.10 (*.35*)

Repetition 2: t(89)= 1.84, p=.07 (*.39*)

Repetition 3: t(89)= 1.01, p=.32 (*.21*)

Baseline differences at visit 2:

Repetition 1: t(89)= 1.68, p=.10 (*.35*)

Repetition 2: t(89)= 1.39, p=.17 (*.29*)

Repetition 3: t(89)= 1.88, p=.06 (*.40*)

The results of the repeated measures ANOVA revealed a significant effect of time; F(14,1246)= 2.13, p=.01 (*.97*), a trending towards significant interaction between treatment*time; F(14,1246)= 2.48, p=<.05 (*.99*), but no significant main effect of treatment; F(1,89)= 1.08, p=.30 (*.18*)

To investigate this significant trend between treatment*time, a one-way ANOVA compared the change-from-baseline performance of those in placebo, to those in resveratrol, at all 15 time-points:

1. F(1,90)= .01, p=.92 (*.00*)

2. F(1,90)= .02, p=.90 (*.00*)

3. F(1,90)= 2.32, p=.13 (*.03*)

4. F(1,90)= 1.82, p=.18 (*.02*)

5. F(1,90)= .46, p=.50 (*.01*)

6. F(1,90)= .04, p=.85 (*.00*)

7. F(1,90)= .26, p=.61 (*.00*)

8. F(1,90)= .37, p=.54 (*.00*)

9. F(1,90)= 4.25, p=.04 (*.05*)

10. F(1,90)= .24, p=.63 (*.00*)

11. F(1,90)= 2.86, p=.09 (*.03*)

12. F(1,90)= 7.22, p=<.01 (*.08*)

13. F(1,90)= 1.96, p=.17 (*.02*)

14. F(1,90)= .16, p=.69 (*.00*)

15. F(1,90)= 7.15, p=<.01 (*.07*)

Three significant effects emerged; at time-point 9 (relating to the visit 2 baseline assessment, specifically the 3^rd^ repetition), where those consuming placebo completed significantly more correct subtractions (2.88 (.64)), compared to resveratrol (.81 (.78)); time-point 12 (relating to the first post-dose assessment during visit 2, specifically the 3^rd^ repetition), where those consuming placebo completed significantly more correct subtractions (3.88 (.74)) compared to resveratrol (.79 (.90)); and at time-point 15 (relating to the second assessment during visit 2, specifically the 3^rd^ repetition), where those consuming placebo completed significantly more correct subtractions (3.08 (.63)) compared to resveratrol (-.05 (1.03)).

#### 7.4.2.3 Errors

Baseline differences at visit 1:

Repetition 1: t(89)= .08, p=.94 (*.02*)

Repetition 2: t(89)= .18, p=.86 (*.04*)

Repetition 3: t(89)= .61, p=.55 (*.13*)

Baseline differences at visit 2:

Repetition 1: t(89)= -.09, p=.93 (*-.02*)

Repetition 2: t(89)= .41, p=.69 (*.09*)

Repetition 3: t(89)= .65, p=.52 (*.14*)

The results of the repeated measures ANOVA revealed no significant effect of time; F(14,1246)= 1.46, p=.12 (*.85*), no significant interaction between treatment*time; F(14,1246)= .63, p=.85 (*.41*), and no significant main effect of time; F(1,89)= .01, p=.91 (*.05*).

### 7.4.3 Rapid Visual Information Processing

#### 7.4.3.1 Percentage Correct

Baseline differences at visit 1:

Repetition 1: t(83)= 2.06, p=.04 (*.45*)

Repetition 2: t(83)= 1.38, p=.17 (*.30*)

Repetition 3: t(83)= 1.45, p=.15 (*.32*)

Baseline differences at visit 2:

Repetition 1: t(83)= 1.41, p=.16 (*.31*)

Repetition 2: t(83)= 1.90, p=.06 (*.41*)

Repetition 3: t(83)= 2.05, p=.04 (*.45*)

Two significant baseline differences were observed. The first was an effect observed on the 1^st^ repetition of the RVIP task during visit 1, where those consuming placebo scored an average of 61.44% (3.04), versus 52.69% (2.93) in the resveratrol condition. This is indicative of a true baseline difference between participants, prior to commencing the trial. The second significant baseline difference was observed on the 3^rd^ repetition of the RVIP task, during visit 2, where those in the placebo condition scored significantly higher (61.56% (3.25)), compared to those in resveratrol (52.38% (3.02)). This would be indicative of a chronic effect of treatment, but the aforementioned baseline difference on day 1 would more likely suggest that this genuine difference between the treatment groups has simply persisted.

The results of the repeated measures ANOVA revealed a significant effect of time; F(14,1162)= 4.37, p=<.01 (*1.00*), a significant interaction between treatment*time; F(14,1162)= .78, p=<.01 (*.51*), but no significant main effect of treatment; F(1,83)= .00, p=.98 (*.05*).

To investigate this significant interaction between treatment*time, a one-way ANOVA compared the change-from-baseline performance of those in placebo, to those in resveratrol, at all 15 time-points:

1. F(1,84)= .32, p=.57 (*.00*)

2. F(1,84)= 2.26, p=.14 (*.03*)

3. F(1,84)= .00, p=.96 (*.00*)

4. F(1,84)= 1.13, p=.29 (*.01*)

5. F(1,84)= .87, p=.35 (*.01*)

6. F(1,84)= 1.78, p=.19 (*.02*)

7. F(1,84)= .55, p=.46 (*.01*)

8. F(1,84)= .92, p=.34 (*.01*)

9. F(1,84)= .58, p=.45 (*.01*)

10. F(1,84)= .03, p=.86 (*.00*)

11. F(1,84)= .21, p=.65 (*.00*)

12. F(1,84)= .08, p=.79 (*.00*)

13. F(1,84)= .13, p=.72 (*.00*)

14. F(1,84)= .33, p=.57 (*.00*)

15. F(1,84)= .08, p=76 (*.00*)

No significant effects were observed on the above post-hoc comparisons.

#### 7.4.3.2 Reaction Time

Baseline differences at visit 1:

Repetition 1: t(83)= .03, p=.98 (*.01*)

Repetition 2: t(83)= .83, p=.41 (*.18*)

Repetition 3: t(83)= 1.44, p=.15 (*.31*)

Baseline differences at visit 2:

Repetition 1: t(83)= .39, p=.70 (*.08*)

Repetition 2: t(83)= .89, p=.38 (*.19*)

Repetition 3: t(83)= .74, p=.46 (*.16*)

The results of the repeated measures ANOVA revealed no significant effect of time; F(14,1162)= .60, p=.87 (*.39*), no interaction between treatment*time; F(14,1162)= .78, p=.70 (*.51*), and no significant main effect of treatment; F(1,83)= .84, p=.36 (*.15*).

#### 7.4.3.3 False Alarms

Baseline differences at visit 1:

Repetition 1: t(83)= -1.69, p=.10 (*-.37*)

Repetition 2: t(83)= -2.17, p=.03 (*-.47*)

Repetition 3: t(83)= -1.90, p=.06 (*-.41*)

Baseline differences at visit 2:

Repetition 1: t(83)= -2.87, p=<.01 (*-.62*)

Repetition 2: t(83)= -2.95, p=<.05 (*-.64*)

Repetition 3: t(83)= -3.08, p=<.05 (*-.67*)

Four significant baseline differences were observed for RVIP false alarms. The first was observed on the 2^nd^ repetition of RVIP during visit 1, where those in the resveratrol condition made significantly more errors (4.25 (.81)) than those in placebo (2.42 (.34)). The remaining 3 were all during the visit 2 baseline, with placebo always making significantly fewer errors than resveratrol; at repetition 1 (2.02 (.36) versus 6.53 (1.62)), repetition 2 (1.89 (.30) versus 5.20 (1.14)), and repetition 3 (1.69 (.28) versus 5.25 (1.19)), respectively. As previously, this naturally superior performance of participants in the placebo condition looks to have persisted into visit 2.

The results of the repeated measures ANOVA revealed a significant effect of time; F(14,1162)= 6.09, p=<.01 (*1.00*), a significant interaction between treatment*time; F(14,1162)= 1.78, p=.04 (*.92*), but no significant main effect of treatment; F(1,83)= 2.29, p=.13 (*.32*).

To investigate this significant interaction between treatment*time, a one-way ANOVA compared the change-from-baseline performance of those in placebo, to those in resveratrol, at all 15 time-points:

1. F(1,84)= .15, p=.71 (*.00*)

2. F(1,84)= .71, p=.40 (*.01*)

3. F(1,84)= .86, p=.36 (*.01*)

4. F(1,84)= .12, p=.73 (*.00*)

5. F(1,84)= .05, p=.82 (*.00*)

6. F(1,84)= 1.83, p=.18 (*.02*)

7. F(1,84)= 2.62, p=.11 (*.03*)

8. F(1,84)= 2.63, p=.11 (*.03*)

9. F(1,84)= 6.21, p=.02 (*.07*)

10. F(1,84)= .00, p=.99 (*.00*)

11. F(1,84)= 2.07, p=.15 (*.02*)

12. F(1,84)= 1.94, p=.17 (*.02*)

13. F(1,84)= .07, p=.79 (*.00*)

14. F(1,84)= 3.88, p=.05 (*.05*)

15. F(1,84)= 4.97, p=.03 (*.06*)

The above post-hoc comparisons revealed 2 significant differences, and 1 trend towards significance. Firstly, at time-point 9 (relating to the 3^rd^ repetition of the baseline assessment during visit 2), participants in the placebo condition significantly reduced their false alarm rate (-.80 (.31)), compared to participants in the resveratrol group (1.25 (.80)), compared to their baseline performance on day 1. The second effect was a trend towards significance (at p=.05), observed at the second repetition of the task at the 2^nd^ post-dose assessment on day 2. Again, those consuming placebo significantly reduced their false alarm rate (-.62 (.33)), as compared to resveratrol, who again showed an increase from the day 1 baseline (1.80 (1.25)). Finally, the 3^rd^ effect was a significant difference at the final repetition of the task, during the 2^nd^ post-dose assessment, at visit 2. Again, placebo participants significantly reduced their false alarm rate (-.58 (.48)), as compared to resveratrol (2.25 (1.23)).

### 7.4.4 Word Recall

Independent samples t tests compared baseline differences on all four word recall outcome measures (immediate word recall correct, immediate word recall incorrect, delayed word recall correct, and delayed word recall incorrect) at visit 1 and visit 2:

Baseline Differences at visit 1:

Immediate Word Recall Correct: t(97)=.02, p=.98 (*-.01*)

Immediate Word Recall Incorrect: t(97)= .81, p=.42 (*-.16*)

Delayed Word Recall Correct: t(97)= .28, p=.78 (*.06*)

Delayed Word Recall Incorrect: t(97)= -2.56, p=.01 (*-.52*)

Baseline Differences at visit 2:

Immediate Word Recall Correct: t(97)= -.70, p=.49 (*-.14*)

Immediate Word Recall Incorrect: t(97)= 1.89, p=.06 (*-.38*)

Delayed Word Recall Correct: t(97)= .31, p=.76 (*-.06*)

Delayed Word Recall Incorrect: t(97)= -1.75, p=.08 (*-.35*)

A significant baseline difference was observed on delayed word recall incorrect during visit 1, where participants in the placebo condition made significantly fewer incorrect responses (.76 (.14)) than resveratrol (1.52 (.26)). As with previous tasks, this represents a natural pre-dose difference between treatment groups.

Repeated measures ANOVA then compared change-from-baseline word recall performance at 5 time-points (visit 1 post-dose assessment 1, visit 1 post-dose assessment 2, visit 2 baseline, visit 2 post-dose assessment 1, and visit 2 post-dose assessment 2):

#### 7.4.4.1 Immediate Word Recall Correct

Time: F(4,388)= 8.97, p=<.01 (*1.00*)

Treatment*Time: F(4,388)= 1.59, p=.18 (*.49*)

Treatment: F(1,97)= .00, p=.95 (*.05*)

#### 7.4.4.2 Immediate Word Recall Incorrect

Time: F(4,388)= .54, p=.71 (*.18*)

Treatment*Time: F(4,388)= .59, p=.67 (*.20*)

Treatment: F(1,97)= .01, p=.94 (*.05*)

#### 7.4.4.3 Delayed Word Recall Correct

Time: F(4,388)= 36.01, p=<.01 (*1.00*)

Treatment*Time: F(4,388)= 1.30, p=.27 (*.41*)

Treatment: F(1,97)= .29, p=.59 (*.08*)

#### 7.4.4.4 Delayed Word Recall Incorrect

Time: F(4,388)= 9.70, p=<.01 (*1.00*)

Treatment*Time: F(4,388)= 1.06, p=.38 (*.33*)

Treatment: F(1,97)= .02, p=.90 (*.05*)

### 7.4.5 Name to Face Recall

Independent sample t tests compared performance between treatments at baseline on all 4 of the name-to-face-recall outcome measures:

Visit 1 Baseline Differences

Percentage Correct: t(95)= -.40, p=.69 (*-.08*)

Reaction Time: t(95)= -1.36, p=.18 (*-.28*)

Percentage Correct Forename: t(95)= -.19, p=.85 (*-.04*)

Percentage Correct Surname: t(95)= -.54, p=.59 (*-.11*)

Visit 2 Baseline Differences

Percentage Correct: t(95)= -.31, p=.76 (*-.06*)

Reaction Time: t(95)= -2.21, p=.03 (*-.45*)

Percentage Correct Forename: t(95)= .77, p=.44 (*.16*)

Percentage Correct Surname: t(95)= -1.24, p=.22 (*-.25*)

A single significant baseline difference was observed at visit 2 for reaction time. Here, those consuming placebo were significantly faster (11243.56 msec (483.69)) than those consuming resveratrol (12683.86 msec (434.18)). As no differences were found at visit 1 baseline, this effect is representative of a true chronic effect of treatment.

Repeated measures ANOVAs then compared change-from-baseline treatment effects at 5 time-points (visit 1 post-dose repetition 1, visit 1 post-dose repetition 2, visit 2 baseline, visit 2 post-dose repetition 1, and visit 2 post-dose repetition 2) on all four name-to-face-recall outcome measures:

#### 7.4.5.1 Percentage Correct

Time: F(4,380)=7.24, p=<.01 (*1.00*)

Treatment*Time: F(4,380)= 2.49, p=.04 (*.71*)

Treatment: F(1,95)= 3.53, p=.06 (*.46*)

#### 7.4.5.2 Reaction Time

Time: F(4,380)= 1.26, p=.29 (*.40*)

Treatment*Time: F(4,380)= .89, p=.47 (*.28*)

Treatment: F(1,95)= .25, p=.62 (*.08*)

#### 7.4.5.3 Percentage Correct Forename

Time: F(4,380)= 4.89, p=<.01 (*.96*)

Treatment*Time: F(4,380)= 1.70, p=.15 (*.52*)

Treatment: F(1,95)= 2.20, p=.14 (*.31*)

#### 7.4.5.4 Percentage Correct Surname

Time: F(4,380)= 3.72, p=<.01 (*.88*)

Treatment*Time: F(4,380)= 2.71, p=.03 (*.75*)

Treatment: F(1,95)= 2.57, p=.11 (*.36*)

Two significant effects were observed here. The first observed a significant interaction between treatment*time for percentage correct. To explore this, a post-hoc 1-way ANOVA compared the effects of treatment at each of the 5 time-points:

1. F(1,96)= 6.32, p=.01 (*.06*)

2. F(1,96)= 6.60, p=.01 (*.07*)

3. F(1,96)= .01, p=.92 (*.00*)

4. F(1,96)= 1.28, p=.26 (*.01*)

5. F(1,96)= 1.54, p=.22 (*.02*)

Two time-points evinced significant effects here, time-points 1 and 2, which relate to the two post-dose assessments on visit 1. In both of these effects, both treatments performed less well compared to baseline on visit 1. However, resveratrol showed a significantly more pronounced reduction at both post-dose repetition 1 (-10.55 (2.39)) compared to placebo (-1.83 (2.50)), and post-dose repetition 2 (-12.77 (2.28)) versus -4.00 (2.52), respectively), suggesting an acute effect of treatment at post-dose visit 1.

The second significant effect was another treatment*time interaction for percentage correct surname. The post-hoc ANOVA observed:

1. F(1,96)= 2.16, p=.15 (*.02*)

2. F(1,96)= 6.92, p=.01 (*.07*)

3. F(1,96)= .64, p=.43 (*.01*)

4. F(1,96)= 2.47, p=.12 (*.03*)

5. F(1,96)= 1.25, p=.27 (*.01*)

The single significant effect here is at time-point 2, relating to the 2^nd^ post-dose assessment during visit 1 and, as above with the overall percentage correct effect, this was due to participants in the resveratrol condition detecting fewer surnames (-13.83 (2.90)), than those in the placebo condition (-3.67 (2.57)). As above, this shows that both treatment groups were detecting fewer correct surnames compared to their baseline performance on visit 1, but resveratrol reduced more significantly.

### 7.4.6 Numeric Working Memory

Visit 1 Baseline Differences

Percentage Correct: t(97)= .44, p=.66 (*.09*)

Reaction Time: t(97)= -.36, p=.72 (*-.07*)

Visit 2 Baseline Differences

Percentage Correct: t(97)= -3.11, p=<.01 (*-.63*)

Reaction Time: t(97)= .16, p=.87 (*.03*)

A significant baseline difference between treatments at visit 2 was observed on the percentage correct outcome measure of Numeric Working Memory, suggesting a pure chronic effect of treatment. Here, resveratrol performed highest, with a mean of 96.58% (.57), compared to the placebo mean of 92.88 (1.02).

Repeated measures ANOVAs then compared treatment effects at the 5 post-visit-1 baseline time-points, on this change-from-baseline data:

#### 7.4.6.1 Percentage Correct

Time: F(4,388)= .95, p=.43 (*.30*)

Time*Treatment: F(4,388)= 5.17, p=<.01 (*.97*)

Treatment: F(1,97)= .75, p=.39 (*.14*)

#### 7.4.6.2 Reaction Time

Time: F(4,388)= 7.95, p=<.01 (*1.00*)

Time*Treatment: F(4,388)= 1.44, p=.22 (*.45*)

Treatment: F(1,97)= .28, p=.60 (*.08*)

A significant interaction between time*treatment was observed for percentage correct, and so post-hoc exploratory t tests compared treatments at all 5 time-points:

1. t(97)= -.92, p=.36 (*-.19*)

2. t(97)= -.61, p=.54 (*-.12*)

3. t(97)= -.23, p=.02 (*-.47*)

4. t(97)= -.16, p=.87 (*-.03*)

5. t(97)= -.02, p=.98 (*-.00*)

This mirrors the above baseline difference, as the only significant post-hoc result was at time-point 3, which relates to the baseline assessment at visit 2. The direction was also the same, with resveratrol reaching a higher change-from-baseline percentage correct (+3.43% (1.81)), compared to placebo (-1.13% (.82)).

### 7.4.7 Choice Reaction Time

Visit 1 Baseline Differences

Percentage Correct: t(97)= -1.07, p=.29 (*-.22*)

Reaction Time: t(97)= -.90, p=.37 (*-.18*)

Visit 2 Baseline Differences

Percentage Correct: t(97)= -.15, p=.88 (*-.03*)

Reaction Time: t(97)= -.32, p=.75 (*-.06*)

Repeated measures ANOVAs comparing change-from-baseline treatment differences across 5 time-points observed:

#### 7.4.7.1 Percentage Correct

Time: F(4,388)= .63, p=.64 (*.21*)

Time*Treatment: F(4,388)= .52, p=.72 (*.17*)

Treatment: F(1,97)= 2.04, p=.16 (*.29*)

#### 7.4.7.2 Reaction Time

Time: F(4,388)= 1.05, p=.38 (*.33*)

Time*Treatment: F(4,388)= .14, p=.97 (*.08*)

Treatment: F(1,97)= .37, p=.54 (*.09*)

### 7.4.8 Corsi Blocks (Span Score)

Visit 1 Baseline Differences: t(97)= .98, p=.33 (*.20*)

Visit 2 Baseline Differences: t(97)= .89, p=.38 (*.18*)

Repeated measures ANOVA on change-from-baseline span score at 5 post-baseline time-points revealed:

#### 7.4.8.1 Span Score

Time: F(4,388)= 2.46, p=.05 (*.70*)

Time*Treatment: F(4,388)= .44, p=.78 (*.15*)

Treatment: F(1,97)= .01, p=.94 (*.05*)

### 7.4.9 Peg and Ball

Visit 1 Baseline Differences

Thinking Reaction Time: t(97)= .54, p=.59 (*.11*)

Completion Reaction Time: t(97)= -.49, p=.62 (*-.10*)

Errors: t(97)= -2.20, p=.03 (*-.44*)

Visit 2 Baseline Differences

Thinking Reaction Time: t(97)= -.88, p=.38 (*-.18*)

Completion Reaction Time: t(97)= -.48, p=.63 (*-.10*)

Errors: t(97)= .60, p=.55 (*.12*)

A significant baseline difference was observed at visit 1, for Peg and Ball errors, representing a true baseline difference between groups. Here, those in the resveratrol condition made significantly more errors on the task, pre-treatment, with a mean of 4.65 (.51) errors, compared to those in the placebo condition (3.25 (.38)).

The repeated measures ANOVA, comparing change-from-baseline performance between treatments at the 5 time-points revealed:

#### 7.4.9.1 Thinking Reaction Time

Time: F(4,388)= 4.31, p=<.01 (*.93*)

Time*Treatment: F(4,388)= 1.51, p=.20 (*.47*)

Treatment: F(1,97)= 3.75, p=.06 (*.48*)

#### 7.4.9.2 Completion Reaction Time

Time: F(4,388)= 4.73, p=<.01 (*.95*)

Time*Treatment: F(4,388)= .84, p=.50 (*.27*)

Treatment: F(1,97)= 1.18, p=.28 (*.19*)

#### 7.4.9.3 Errors

Time: F(4,388)= .51, p=.73 (*.17*)

Time*Treatment: F(4,388)= 1.20, p=.31 (*.38*)

Treatment: F(1,97)= 1.20, p=.28 (*.19*)

### 7.4.10 Picture Recognition

Visit 1 Baseline Differences

Percentage Correct: t(97)= -.91, p=.37 (*-.18*)

Reaction Time: t(97)= .81, p=.42 (*.16*)

Visit 2 Baseline Differences

Percentage Correct: t(97)= -.64, p=.53 (*-.13*)

Reaction Time: t(97)= .33, p=.74 (*.07*)

The repeated measures ANOVA, comparing change-from-baseline performance between treatments at the 5 time-points revealed:

#### 7.4.10.1 Percentage Correct

Time: F(4,388)= 12.22, p=<.01 (*1.00*)

Time*Treatment: F(4,388)= 1.95, p=.10 (*.59*)

Treatment: F(1,97)= .38, p=.54 (*.09*)

#### 7.4.10.2 Reaction Time

Time: F(4,388)= .84, p=.50 (*.27*)

Time*Treatment: F(4,388)= 1.88, p=.11 (*.57*)

Treatment: F(1,97)= .95, p=.33 (*.16*)

### 7.4.11 Word Recognition

Visit 1 Baseline Differences

Percentage Correct: t(97)= -.15, p=.88 (*-.03*)

Reaction Time: t(97)= -.45, p=.65 (*-.09*)

Visit 2 Baseline Differences

Percentage Correct: t(97)= -.15, p=.88 (*-.03*)

Reaction Time: t(97)= -1.06, p=.29 (*-.21*)

The repeated measures ANOVA, comparing change-from-baseline performance between treatments at the 5 time-points revealed:

#### 7.4.11.1 Percentage Correct

Time: F(4,388)= 12.80, p=<.01 (*1.00*)

Time*Treatment: F(4,388)= 1.87, p=.12 (*.57*)

Treatment: F(1,97)= 1.41, p=.24 (*.22*)

#### 7.4.11.2 Reaction Time

Time: F(4,388)= 2.61, p=.04 (*.73*)

Time*Treatment: F(4,388)= 1.03, p=.39 (*.33*)

Treatment: F(1,97)= .09, p=.76 (*.06*)

## **7.5. Mood**

### 7.5.1 Profile Of Mood States (POMS)

An independent sample t test first compared the raw mood scores of placebo and resveratrol at visit 1 (to ascertain whether any pre-existing mood differences existed between treatment groups, prior to any intervention), and visit 2 (to ascertain whether a chronic effect of treatment had taken place):

Visit 1 Baseline Differences:

Tension-Anxiety: t(96)= 1.58, p=.12 (*-.32*)

Depression-Dejection: t(96)= -2.03, p=.05 (*-.41*)

Anger-Hostility: t(96)= -1.12, p=.26 (*-.23*)

Vigour-Activity: t(96)= .39, p=.70 (*.08*)

Fatigue-Inertia: t(96)= -1.65, p=.10 (*-.33*)

Confusion-Bewilderment: t(96)= -1.96, p=.05 (*-.40*)

Friendliness: t(96)= -.95, p=.34 (*-.19*)

Total Mood Disturbance: t(96)= -1.77, p=.08 (*-.36*)

Visit 2 Baseline Differences:

Tension-Anxiety: t(96)= -1.21, p=.23 (*-.24*)

Depression-Dejection: t(96)= -2.01, p=.05 (*-.41*)

Anger-Hostility: t(96)= -2.35, p=.02 (*-.47*)

Vigour-Activity: t(96)= -.16, p=.87 (*-.03*)

Fatigue-Inertia: t(96)= -.63, p=.53 (*-.13*)

Confusion-Bewilderment: t(96)= -.48, p=.63 (*-.10*)

Friendliness: t(96)= -1.29, p=.20 (*-.26*)

Total Mood Disturbance: t(96)= -1.00, p=.32 (*-.20*)

Only 1 significant baseline difference was observed, and this revealed that those in the placebo condition reported a significantly smaller change in anger/hostility at visit 2 (.60 (.16)), as compared to those in resveratrol (1.48 (.35)). With no effect observed here at pre-dose on visit 1, this represents a chronic effect of treatment at visit 2.

A repeated measures ANOVA then compared change-from-baseline mood, between the 2 treatment conditions, at 3 time-points (visit 1 post-dose, visit 2 baseline, and visit 2 post-dose). No significant treatment effects were observed on any POMS mood measure:

#### 7.5.1.1 Tension-Anxiety

Time: F(2,192)= 13.20, p=<.01 (*1.00*)

Treatment*Time: F(2,192)= .58, p=.56 (*.15*)

Treatment: F(1,96)= .10, p=.75 (*.06*)

#### 7.5.1.2 Depression-Dejection

Time: F(2,192)= 10.51, p=<.01 (*.99*)

Treatment*Time: F(2,192)= .70, p=.50 (*.17*)

Treatment: F(1,96)= .65, p=.42 (*.13*)

#### 7.5.1.3 Anger-Hostility

Time: F(2,192)= .78, p=.46 (*.18*)

Treatment*Time: F(2,192)= .27, p=.76 (*.09*)

Treatment: F(1,96)= 1.23, p=.27 (*.20*)

#### 7.5.1.4 Vigour-Activity

Time: F(2,192)= 68.26, p=<.01 (*1.00*)

Treatment*Time: F(2,192)= .16, p=.86 (*.07*)

Treatment: F(1,96)= .57, p=.45 (*.12*)

#### 7.5.1.5 Fatigue-Inertia

Time: F(2,192)= 98.51, p-<.01 (*1.00*)

Treatment*Time: F(2,192)= .01, p=1.00 (*.05*)

Treatment: F(1,96)= 1.06, p=.31 (*.17*)

#### 7.5.1.6 Confusion-Bewilderment

Time: F(2,192)= 67.53, p=<.01 (*1.00*)

Treatment*Time: F(2,192)= .02, p=.98 (*.05*)

Treatment: F(1,96)= 3.76, p=.06 (*.48*)

#### 7.5.1.7 Friendliness

Time: F(2,192)= 34.98, p=<.01 (*1.00*)

Treatment*Time: F(2,192)= .68, p=.51 (*.16*)

Treatment: F(1,96)= .10, p=.75 (*.06*)

#### 7.5.1.8 Total Mood Disturbance

Time: F(2,192)= 73.78, p=<.01 (*1.00*)

Treatment*Time: F(2,192)= .16, p=.85 (*.07*)

Treatment: F(1,96)= 1.13, p=.29 (*.18*)

### 7.5.2 Bond Lader (completed during COMPASS task battery)

Visit 1 Baseline Differences:

Alert: t(97)= .72, p=.48 (*.14*)

Content: t(97)= .24, p=.81 (*.05*)

Calm: t(97)= 1.79, p=.08 (*.36*)

Visit 2 Baseline Differences:

Alert: t(97)= .85, p=.40 (*.17*)

Content: t(97)= .94, p=.35 (*.19*)

Calm: t(97)= .47, p=.64 (*.10*)

Repeated measures ANOVAs comparing treatment differences on change-from-baseline data at 5 timepoints:

#### 7.5.2.1 Alert

Time: F(4,388)= 2.98, p=.02 (*.79*)

Time*Treatment: F(4,388)= .13, p=.97 (*.08*)

Treatment: F(1,97)= .55, p=.46 (*.11*)

#### 7.5.2.2 Content

Time: F(4,388)= 5.24, p=<.01 (*.97*)

Time*Treatment: F(4,388)= .40, p=.81 (*.14*)

Treatment: F(1,97)= .13, p=.72 (*.07*)

#### 7.5.2.3 Calm

Time: F(4,388)= 3.27, p=.01 (*.83*)

Time*Treatment: F(4,388)= 1.14, p=.34 (*.36*)

Treatment: F(1,97)= 3.12, p=.08 (*.42*)

### 7.5.3 Visual Analogue Scales (completed during COMPASS task battery)

Visit 1 Baseline Differences:

Task Difficulty

Repetition 1: t(97)= -1.41, p=.16 (*-.28*)

Repetition 2: t(97)= -.90, p=.37 (*-.18*)

Repetition 3: t(97)= -1.64, p=.11 (*-.33*)

Mental Fatigue

Repetition 1: t(97)= -.13, p=.89 (*-.03*)

Repetition 2: t(97)= -.85, p=.40 (*-.17*)

Repetition 3: t(97)= -1.52, p=.13 (*-.31*)

Visit 2 Baseline Differences:

Task Difficulty

Repetition 1: t(97)= -.96, p=.34 (*-.19*)

Repetition 2: t(97)= -1.16, p=.25 (*-.23*)

Repetition 3: t(97)= -1.35, p=.18 (*-.27*)

Mental Fatigue

Repetition 1: t(97)= -.84, p=.40 (*-.17*)

Repetition 2: t(97)= -1.66, p=.10 (*-.33*)

Repetition 3: t(97)= -1.40, p=.16 (*-.28*)

The Visual Analogue Scales were repeated 3 times during the COMPASS tasks, following each of the 3 repetitions of the Cognitive Demand Battery (Serial 3 and 7 Subtractions, and the Rapid Visual Information Processing task) and, as such, repeated measures ANOVAs compared treatment differences at 15 time-points.

#### 7.5.3.1 Task Difficulty

Time: F(14,1358)= 5.90, p=<.01 (*1.00*)

Time*Treatment: F(14,1358)= .64, p=.83 (*.42*)

Treatment: F(1,97)= .13, p=.72 (*.07*)

#### 7.5.3.2 Mental Fatigue

Time: F(14,1358)= 21.66, p=<.01 (*1.00*)

Time*Treatment: F(14,1358)= 1.04, p=.41 (*.67*)

Treatment: F(1,97)= .51, p=.48 (*.11*)

## **7.6. Summary of Significant Effects**

##### **Table 7.6.1. Summary of Significant Study Outcomes**

| **Outcome Measure** | **Significance** | **Description** |
| --- | --- | --- |
| **Cholesterol** | A main effect of treatment was observed; F(1,15)= 5.10, p=.04 (*.56*), with those in placebo showing the greatest reduction in cholesterol levels overall (mean; -25.24, SE; 15.67), compared to resveratrol (mean; 23.39, SE; 14.78). However, those in the placebo condition began the study with significantly higher cholesterol (mean; 203.52 mmol/L, SD; 44.88) than those in resveratrol (mean; 140.34 mmol/L, SD; 13.88); t(15)=4.03, p=<.01 (*1.96*). | Statistically, challenging to interpret the main effect of treatment, due to existing baseline differences.  However, **placebo** participants began the trial with significantly higher cholesterol than those in resveratrol, and they showed a significant lowering of this at the visit 2 time-point. Thus, one could argue, that they had the capacity to show this reduction. |
| **Heart Rate** | A significant main effect of treatment was observed; F(1,96)= 7.88, p=<.05 (*.79*). Here, heart rate was significantly lower in the placebo condition (mean change; -1.37 (SE; .72)), compared to resveratrol (mean change; 1.55 (SE; .75)). | Heart Rate was significantly lower, overall, in the **placebo** condition, compared to resveratrol. With no pre-dose, visit 1 effect, this can be interpreted as a true effect of treatment, observed across both visit 1 and 2, relative to the visit 1 baseline. |
| **Serial 3 Subtractions**  **‘Total’** | Significant baseline differences were observed at visit 2:  Rep 1: t(92)= 2.05, p=.04 (*.42*)  Rep 2: t(92)= 2.39, p=.02 (*.49*)  Rep 3: t(92)= 2.44, p=.02 (*.50*)  Where placebo outperformed resveratrol at all 3 repetitions:  Rep 1: 36.15 (2.08) versus 30.55 (1.78)  Rep 2: 39.47 (2.07) versus 32.96 (1.78)  Rep 3: 40.57 (2.03) versus 34.11 (1.70)  However, because there was a significant difference between treatments at the visit 1 (pre-dose) baseline (at repetition 3 only: t(92)= 2.19, p=.03 (.45), but the others were trending), this would suggest that a true difference existed between the treatment groups pre-intervention, and so the visit 2 effects are probably just a continuation of this, rather than a pure chronic effect of treatment. | No real interpretable difference due to existing difference between groups at pre-dose visit 1. |
| **Serial 3 Subtractions ‘Correct’** | As above for ‘total’, significant differences were seen during baseline at visit 2:  Rep 1: t(92)= 2.37, p=.02 (.49)  Rep 2: t(92)= 2.59, p=.01 (.53)  Rep 3: t(92)= 2.52, p=.01 (.52)  Where placebo outperformed resveratrol at all 3 repetitions:  Rep 1: 34.85 (2.11) versus 28.34 (1.75)  Rep 2: 37.66 (2.14) versus 30.26 (1.89)  Rep 3: 38.68 (2.10) versus 31.79 (1.76)  However, there was again a baseline difference on this measure at visit 1, repetition 3 (t(92)= 2.14, p=.04 (.44)), with the others trending towards significance also. Again, likely a persistence of a true difference between groups, rather than a chronic effect of treatment. | No real interpretable difference due to existing difference between groups at pre-dose visit 1. |
| **Serial 3 Subtractions ‘Errors’** | A significant effect (and a trend towards significance) were observed on the first 2/3 repetitions during visit 2:  Rep 1: t(92)= -2.17, p=.03 (-.45)  Rep 2: t(92)= -1.91, p=.06 (-.39)  At repetition 1, participants consuming placebo made approximately 1 fewer errors (1.30 (.25)), compared to those consuming resveratrol (2.21 (.34)). And this pattern was the same for repetition 2. | A significant reduction in errors pre-dose at visit 2 (compared to visit 1 performance), for those consuming **placebo.** This effect didn’t persist into visit 2 though, once treatment was consumed, so this can be interpreted as a pure chronic effect of treatment only. |
| **Serial 7 Subtractions**  **‘Total’** | A significant baseline difference was observed on the 3rd, and final, baseline repetition on visit 2: t(89)= 2.06, p=.04 (.43). Here, participants in the placebo condition completed significantly more subtractions (26.84 (1.56)) than those consuming resveratrol (22.31 (1.53)). This represents a chronic effect of treatment.  Additionally, a significant interaction between treatment*time; F(14,1246)= 2.55, p=<.05 (.99) was observed on the repeated measures ANOVA which, when explored further, showed significant differences between treatment at repetitions:  9. F(1,90)= 4.55, p=.04 (.05)  12. F(1,90)= 4.42, p=.04 (.05)  15. F(1,90)= 5.75, p=.02 (.06)  Interestingly, these time-points relate to the 3^rd^, and final, repetitions of the task during visit 2 (9 being the baseline, and 12 and 15 being the two post-dose assessments). In all cases, placebo outperformed resveratrol; (2.39 (.61)) versus .31 (.77); 3.02 (.65)) versus .98 (.73); 2.76 (.62) versus.19 (.90), respectively). | A pure chronic effect of treatment at visit 2, where **placebo** participants completed more Serial 7 Subtractions than resveratrol.  Effect was supported by an acute effect of treatment within visit 2, where **placebo** participants completed more Serial 7 Subtractions at all of the 3 repetitions of the task.  Interestingly both the pure chronic, and acute effect of treatment within visit 2, were all on the 3^rd^ and final repetition of the Serial 7s task, which might suggest a treatment effect manifesting during the most demanding repetition of the task, when it is most required. |
| **Serial 7 Subtractions ‘Correct’** | A trending towards significant interaction between treatment*time; F(14,1246)= 2.48, p=<.05 (.99) was observed. Post hoc analysis observed significant differences at the same repetitions as the above ‘total’ outcome:  9. F(1,90)= 4.25, p=.04 (.05)  12. F(1,90)= 7.22, p=<.01 (.08)  15. F(1,90)= 7.15, p=<.01 (.07)  As above, placebo completed more correct Serial 7 Subtractions than resveratrol (2.88 (.64) versus .81 (.78); 3.88 (.74) versus .79 (.90); and 3.08 (.63) versus -.05 (1.03), respectively). | A trending towards significant interaction between treatment*time was observed within visit 2, where **placebo** participants completed more correct Serial 7 Subtractions at all of the 3 repetitions of the task.  This was an acute effect of treatment within visit 2 only. Like Serial 7s total above, this was seen at all 3 repetitions at visit 2 but, we saw no pure chronic effect of treatment at visit 2 here. |
| **RVIP**  **‘% Correct’** | Significant baseline differences were seen at visit 1; Rep 1: t(83)= 2.06, p=.04 (.45), and at visit 2; Rep 3: t(83)= 2.05, p=.04 (.45), with placebo outperforming resveratrol in both cases; 61.44% (3.04), versus 52.69% (2.93) and 61.56% (3.25) versus 52.38% (3.02), respectively. This suggests than a naturally superior ability in the placebo condition persisted into visit 2.  A significant interaction between treatment*time was observed; F(14,1162)= .78, p=<.01 (.51), but none of the post-hoc comparisons at the 15 time-points revealed any significant treatment differences. | No real interpretable difference due to existing difference between groups at pre-dose visit 1. |
| **RVIP**  **‘False Alarms’** | Significant baseline differences were seen during visit 1:  Rep 2: t(83)= -2.17, p=.03 (-.47)  And visit 2:  Rep 1: t(83)= -2.87, p=<.01 (-.62)  Rep 2: t(83)= -2.95, p=<.05 (-.64)  Rep 3: t(83)= -3.08, p=<.05 (-.67)  Where those in the placebo condition made significantly fewer errors at visit 1; 2.42 (.34) versus 4.25 (.81), and all 3 repetitions at visit 2; 2.02 (.36) versus 6.53 (1.62), 1.89 (.30) versus 5.20 (1.14)), and 1.69 (.28) versus 5.25 (1.19)), respectively. This suggests that an existing superior ability in the placebo condition persisted into visit 2.  A significant interaction between treatment*time was also observed; F(14,1162)= 1.78, p=.04 (.92) which, when interrogated further, revealed significant (plus 1 trend) treatment differences at the following time-points:  9. F(1,84)= 6.21, p=.02 (.07)  14. F(1,84)= 3.88, p=.05 (.05)  15. F(1,84)= 4.97, p=.03 (.06)  Relative to visit 1 baseline, participants in the placebo condition significantly reduced their false alarm rate in all 3 cases; (-.80 (.31) versus 1.25 (.80); -.62 (.33) versus 1.80 (1.25); and -.58 (.48) versus 2.25 (1.23), respectively). | No real interpretable difference due to existing difference between groups at pre-dose visit 1. |
| **Name-to-Face Recall**  **‘Reaction Time’** | A significant baseline difference was observed at visit 2: t(95)= -2.21, p=.03 (-.45). Here, those consuming placebo were significantly faster (11243.56 msec (483.69)) than those consuming resveratrol (12683.86 msec (434.18)). As no differences were found at visit 1 baseline, this effect is representative of a true chronic effect of treatment. | **Placebo** participants performed more quickly at the pre-dose assessment at visit 2, suggesting a pure chronic effect of treatment. |
| **Name-to-Face Recall**  **‘% Correct’** | A significant interaction between treatment*time was observed; F(4,380)= 2.49, p=.04 (.71). A post-hoc 1-way ANOVA found significant treatment differences at time-points:  1. F(1,96)= 6.32, p=.01 (.06)  2. F(1,96)= 6.60, p=.01 (.07)  Which relate to the 2 post-dose assessments during visit 1; i.e. acute effects of treatment within visit 1.  In both of these effects, both treatments performed less well compared to baseline on visit 1. However, resveratrol showed a significantly more pronounced reduction at both post-dose repetition 1 (-10.55 (2.39)) compared to placebo (-1.83 (2.50)), and post-dose repetition 2 (-12.77 (2.28)) versus -4.00 (2.52), respectively). | **Placebo** showed an attenuation of reduced performance between the visit 1 baseline, to both of the visit 1 post-dose assessments. This is an acute effect of treatment within day 1. |
| **Name-to-Face Recall**  **‘% Correct Surname’** | A significant interaction between treatment*time was observed; F(4,380)= 2.71, p=.03 (.75). The post-hoc ANOVA found a significant treatment difference at only time-point 2 (i.e. the 2^nd^ post-dose assessment during visit 1; F(1,96)= 6.92, p=.01 (.07).  This was due to participants in the resveratrol condition detecting fewer surnames (-13.83 (2.90)), than those in the placebo condition (-3.67 (2.57)). As above, this shows that both treatment groups were detecting fewer correct surnames compared to their baseline performance on visit 1, but resveratrol reduced more significantly. | **Placebo** showed an attenuation of reduced performance between the visit 1 baseline, to both of the visit 1 post-dose assessments. This is an acute effect of treatment within day 1. |
| **Numeric Working Memory**  **‘% Correct’** | A significant baseline difference between treatments at visit 2 was observed; t(97)= -3.11, p=<.01 (-.63), suggesting a pure chronic effect of treatment. Here, resveratrol performed highest, with a mean of 96.58% (.57), compared to the placebo mean of 92.88 (1.02).  A significant interaction between time*treatment was observed; F(4,388)= 5.17, p=<.01 (.97), and post-hoc exploratory t tests found a single significant effect at time-point 3 (baseline assessment during visit 2); t(97)= -.23, p=.02 (-.47). This mirrors the above baseline difference, as the only significant post-hoc result was at time-point 3, which relates to the baseline assessment at visit 2. The direction was also the same, with resveratrol reaching a higher change-from-baseline percentage correct (+3.43% (1.81)), compared to placebo (-1.13% (.82)). | **Resveratrol** performed better at baseline during visit 2, suggesting a pure chronic benefit of resveratrol on successful completion of the NWM task. |
| **Profile Of Mood States**  **‘Anger-Hostility’** | A significant baseline difference during visit 2; t(96)= -2.35, p=.02 (-.47) showed that those in the placebo condition reported a significantly smaller change in anger/hostility at visit 2 (.60 (.16)), as compared to those in resveratrol (1.48 (.35)). | **Placebo** participants reported a smaller increase in anger/hostility at visit 2. With no effect observed here at pre-dose on visit 1, this represents a chronic effect of treatment at visit 2. |

* In the Description column, the positive treatment is identified in bold

## **7.6.2 Results Summary**

In summary, participants consuming placebo were observed to have significantly lower cholesterol at visit 2, as compared to visit 1. As they also enrolled on the trial with significantly higher cholesterol, pre-intervention, at visit 1, this could suggest that these participants had a greater requirement for lowered cholesterol when commencing the intervention.

Participants consuming placebo also revealed significantly lowered heart rate during both visit 1, and 2, relative to pre-dose baseline on visit 1. This would suggest that an acute treatment-related lowering of heart rate had taken place during both visits.

Clear significant improvements were also seen in response to placebo for Serial 3 Subtractions ‘errors’; where a lowering of error responses was observed pre-dose at visit 2, suggesting a pure chronic effect of treatment. Serial 7 Subtractions ‘total’ and ‘correct’ responses were also improved in the placebo condition. Here, acute treatment-related improvements were observed during both visit 1 and 2, but ‘total’ responses also revealed a pure chronic improvement, before treatment was consumed, at visit 2. On the Name-to-Face Recall task, placebo participants performed more quickly pre-dose at visit 2, suggesting a pure chronic increase in speed and, during visit 1, while both treatment groups reduced their percentage accuracy in detecting names, and surnames, from baseline, this was significantly attenuated at both post-dose time-points for placebo, suggesting an acute effect of treatment within visit 1 here. Both treatment groups also showed an increase in ‘anger-hostility’ (on the Profile Of Mood States questionnaire) between visit 1 and visit 2 but, again, this was significantly tempered in the placebo condition.

Some further significant positive effects were attributed to placebo, but these are perhaps harder to confidently interpret, as they exist alongside significant baseline differences between the treatment groups upon enrolment, i.e. before the intervention commenced. These outcomes comprise; Serial 3 Subtraction performance (‘total’ and ‘correct’ responses), and Rapid Visual Information Processing performance (‘percentage correct’ and ‘false alarms’). In the case of Serial 3 Subtraction performance, significant pure chronic effects of placebo were observed pre-dose at visit 2. However, baseline differences were already apparent during baseline at visit 1. This was also the case for Rapid Visual Information Processing ‘percentage correct’ performance. Additionally, for Rapid Visual Information Processing ‘false alarm’ rate, placebo participants significantly improved their performance here across the post-dose period during visit 2.

Finally, Numeric Working Memory ‘percentage correct’ and Rapid Visual Information Processing ‘false alarms’ (completed during the cerebral blood flow assessment) both evinced effects in favour of resveratrol. The former outcome revealed a better percentage correct detection for resveratrol participants at baseline during visit 2, suggesting a pure chronic effect of treatment, and resveratrol participants made significantly fewer false alarms on the RVIP task during visit 2, compared to placebo.

To conclude, the existence of extensive pre-intervention differences between the treatment groups hugely hinders interpretation of the findings. Even those aforementioned tasks without significant baseline differences are called into question, because those consuming placebo showed such a profound naturally superior cognitive ability before treatment commenced, that this would likely have impacted all aspects of cognitive function, even if this wasn’t detected statistically.
